# Microreactor equipped with naturally acid-resistant histidine ammonia lyase from an extremophile†

**DOI:** 10.1039/d2ma00051b

**Published:** 2022-03-29

**Authors:** Carina Ade, Thaís F. Marcelino, Mark Dulchavsky, Kevin Wu, James C. A. Bardwell, Brigitte Städler

**Affiliations:** a Interdisciplinary Nanoscience Center (iNANO), Aarhus University, Gustav Wieds Vej 14, Aarhus, 8000, Denmark.; b Sino-Danish Center for Education and Research, University of Chinese Academy of Sciences, China; c Department of Molecular, Cellular, and Developmental Biology and Howard Hughes Medical Institute, University of Michigan, Ann Arbor, MI, 48109, USA.

## Abstract

Extremophile enzymes are useful in biotechnology and biomedicine due to their abilities to withstand harsh environments. The abilities of histidine ammonia lyases from different extremophiles to preserve their catalytic activities after exposure to acid were assessed. *Thermoplasma acidophilum* histidine ammonia lyase was identified as an enzyme with a promising catalytic profile following acid treatment. The fusion of this enzyme with the maltose-binding protein or co-incubation with the chaperone HdeA further helped *Thermoplasma acidophilum* histidine ammonia lyase to withstand acid treatments down to pH 2.8. The assembly of a microreactor by encapsulation of MBP-*Thermoplasma acidophilum* histidine ammonia lyase into a photocrosslinked poly(vinyl alcohol) hydrogel allowed the enzyme to recover over 50% of its enzymatic activity following exposure to simulated gastric and intestinal fluids. Our results show that using engineered proteins obtained from extremophiles in combination with polymer-based encapsulation can advance the oral formulations of biologicals.

## Introduction

Enzymes can catalyze reactions with unparalleled speed and specificity. For this reason, they have been employed as precisely targeted therapeutics to control the levels of specific metabolites or toxins.^1-6^ However, in order to achieve their desired effects, enzymes must remain intact until they reach the location where their activity is required. Oral delivery is generally the preferred route of drug administration to humans, in part because its low invasiveness results in good patient compliance. A significant barrier to oral enzyme therapies is the gastrointestinal tract, a harsh environment that requires enzymes to be resistant to both the low pH conditions and proteases that are present there. Although encapsulation in nano- or microcarriers, such as liposomes or hydrogels, may enhance enzyme stability and confer protection against proteases,^8,9^ encapsulation alone may be insufficient to overcome the challenge presented by low pH. Therefore, enzymes delivered orally also need to be very robust.

Extremophiles are organisms that live and thrive in extreme conditions, such as at high temperatures, high salinities, or high or low pH levels.^10,11^ Enzymes expressed by extremophiles are sometimes referred to as extremozymes.^12^ Extremozymes have qualities that make them preferable in some biotechnology applications, ranging from the food industry to textiles or bioremediation and biofuels, and these have been summarized in multiple reviews.^11-14^ Taq polymerase from *Thermophilus aquaticus* is an example of a widely used extremozyme from a thermophile that has been extensively exploited in PCR for DNA amplification.^15^ Acidophilic extremozymes can be employed for starch processing, desulfurization of coal, recovery of valuable metals, or reduction of heavy metal ions.^11-13^ In addition, extremozymes are used in pharmaceutical settings, *e.g.*, for the production of pharmaceutically relevant molecules such as antibiotics or anti-inflammatory serine protease inhibitors.^16,17^

Though organisms thriving in extreme environments may at first glance seem to be more likely to have developed proteins robust to the stresses of their environments, the growth conditions of these organisms are not necessarily reflected in the properties of their purified enzymes. For instance, although acidophiles have low optimal pH for growth and proliferation, they often maintain their cytosolic pH at levels above the environmental pH through the use of impermeable membranes, proton efflux pumps and by the buffering effects of their cytosolic contents, thereby relieving at least some pressure for the cytosolic enzymes to be acid resistant.^11,18^ It is, therefore, difficult to guarantee that enzymes isolated from organisms that grow under acidic conditions have an intrinsic resistance to acid stress. It is also a challenge to predict *de novo*, merely through sequence or structural analysis, what proteins may be acid tolerant. Previous studies have identified some common features of acidophilic enzymes, including fewer basic amino acids and more aspartic and glutamic acids on their surfaces compared to proteins that are not acid stable.^13,19^ The acidic amino acids tend to be neutral at low pH, whereas basic amino acids are positively charged, which could lead to repulsion and partial protein unfolding.^13,19,20^ Thermophilic extremozymes in general are more densely packed than mesophile enzymes, resulting in their smaller surface area to size ratios and, therefore, their more controlled interactions with solvents and greater stability.^12,13,21^

Here, we aimed to exploit the evolutionary adaptations of extremophile’s enzymes to formulate an acid-resistant enzyme microreactor that can withstand the harsh conditions of the gastrointestinal tract. We decided to use the enzyme histidine ammonia lyase (HAL), an enzyme from a family with broad synthetic and therapeutic applications, as a test case.^22,23^ HAL is a tetrameric enzyme that catalyzes the first step in histidine catabolism and thus is important for central metabolism; consequently, it exists in a wide variety of organisms from across the tree of life.^24^

Specifically, we demonstrate that the HAL enzyme originating from the archaeon *Thermoplasma acidophilum* is tolerant to both heat and acid stresses. We encapsulated this enzyme by various methods, and we found that formulations of the enzyme within hydrogels provided the best resistance to protease digestion. Our strategy of using acid tolerant orthologs of therapeutically relevant enzymes, then encapsulating them to protect them from proteases may provide an effective platform for the oral delivery of protein therapeutics.

## Results and discussion

### Expression and activities of histidine ammonia lyases (HALs) from different extremophiles

Six different organisms were selected based on their acid and heat resistances and how well studied the organisms are, as judged by the total numbers of scientific publications on proteins derived from those organisms. We focused on organisms that are both acid tolerant and heat tolerant in part because heat-tolerant enzymes are often acid tolerant.^20^ We selected the organisms *Acidilobus saccharovorans*, *Caldisphaera lagunensis*, *Alicyclobacillus acidocaldarius*, *Picrophilus torridus*, *Kosmotoga olearia*, and *Thermoplasma acidophilum* for further study. The optimal growth pH and temperatures for these organisms are shown in ESI,† Table S1.^25-34^ All of these organisms possess the enzyme histidine ammonia lyase (HAL), the target of our study.

Versions of HAL genes from the acidophilic thermophiles listed above were codon optimized for expression in *E. coli* and synthesized by Genscript (New Jersey, USA). Each HAL gene was received, cloned into a pUC57 vector, then subcloned into pET28 expression vectors with a His-SUMO N-terminal fusion to enable purification and transformed into *E. coli* strains to determine enzyme expression quality.

We first investigated the expression of the HAL genes from *Acidilobus saccharovorans* and *Caldisphaera lagunensis*, as these organisms are from a group of archaea that are thought to commonly allow disulfide bond formation within their cytosolic proteins.^28^ Naturally occurring disulfides often enhance a protein’s overall stability.^35^ *Acidilobus saccharovorans* HAL and *Caldisphaera lagunensis* HAL proteins contain conserved cysteine residues that are close in three dimensional space in their predicted protein structures and thus appear capable of forming disulfide bonds. However, neither expression in the periplasmic compartment, where disulfide bond formation occurs naturally, nor cytosolic expression of these proteins in two different *E. coli* expression strains optimized for disulfide bond formation in the cytosolic compartment (SHuffle K12 and SHuffle B) resulted in significant amounts of soluble HAL protein. This was independent of the expression temperature, the amount of inducer, or the presence of fusions with the maltose-binding protein (MBP), which is commonly used as a solubility tag.^36^

**Fig. 1:**
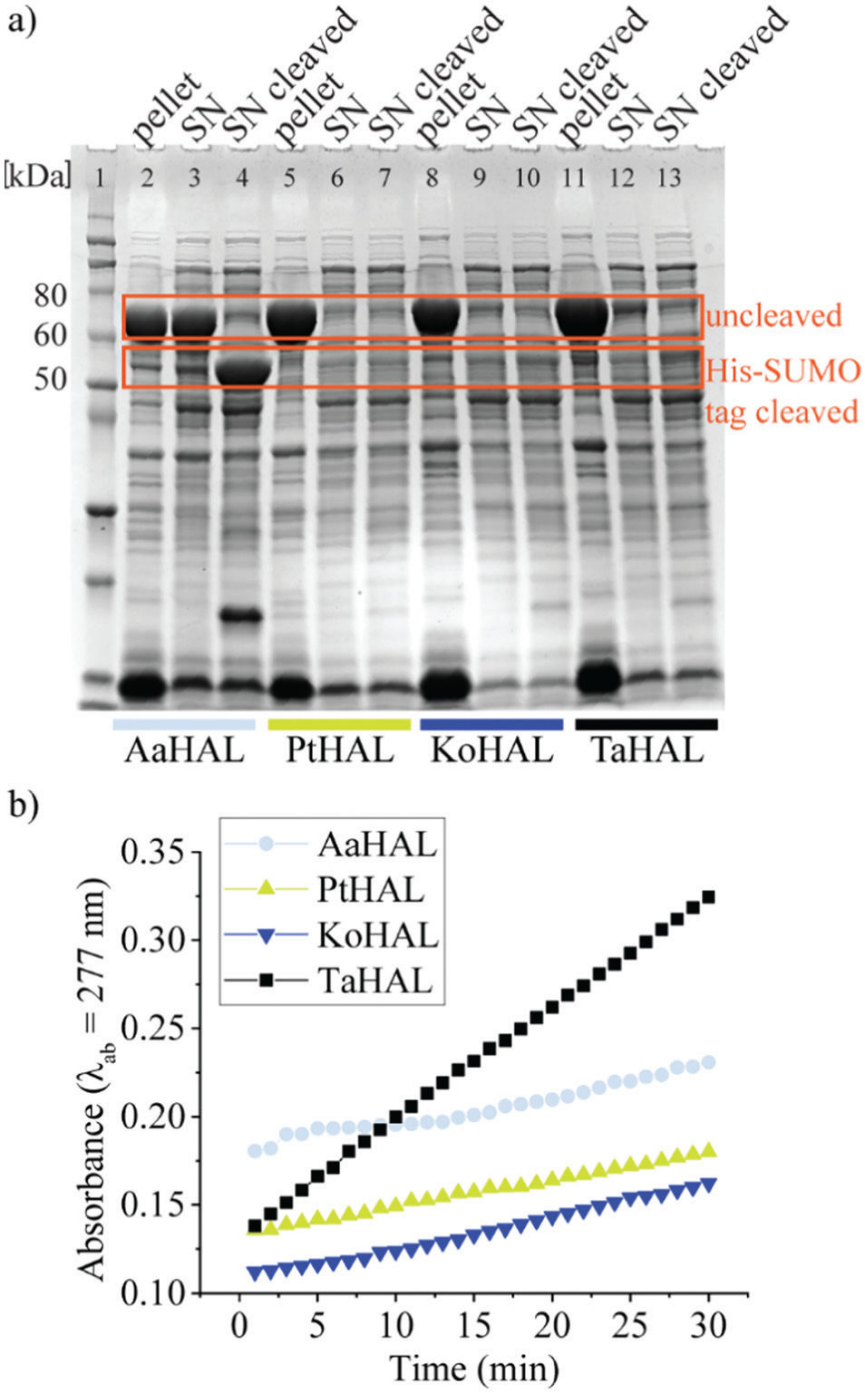
Expression of HALs of extremophiles in *E. coli*. (a) Coomassiestained SDS-PAGE of the expression profiles from pET28a in cell lysate separated into pellet, supernatant (SN) and supernatant after cleavage of the His-SUMO tag (SN cleaved) for *Alicyclobacillus acidocaldarius* (AaHAL, lane 2–4), *Picrophilus torridus* HAL (PtHAL, lane 5–7), *Kosmotoga olearia* HAL (KoHAL, lane 8–10), and *Thermoplasma acidophilum* HAL (TaHAL, lane 11–13). Lane 1: Novex™ Sharp Pre-stained Protein Standard. (b) The activity of the cell lysates before cleavage of the His-SUMO tag measured *via* the absorbance (*λ*_ab_ = 277 nm) of the product *trans*-urocanic acid when incubated with 0.05 mM histidine.

We then moved on to testing HAL enzymes from other acidothermophiles. Although most organisms maintain their cytosolic pH at near neutral, there are several organisms that allow their intracellular pH to drop substantially when they are exposed to acidic growth conditions. *Thermoplasma acidophilum*, for instance, has a cytosolic pH of 5.5 when grown at a pH between 1 and 2.^32,37^ *Thermoplasma acidophilum* is therefore of special interest because its proteins are particularly likely to be able to withstand low pH values without denaturing irreversibly. *Thermoplasma acidophilum* HAL, *Kosmotoga olearia* HAL, *Alicyclobacillus acidocaldarius* HAL, and *Picrophilus torridus* HAL were expressed from a pET28a vector containing an N-terminal His-SUMO tag. All of these enzymes were well expressed as fusion proteins, as indicated by the strong bands at approximately 70 kDa on the sodium dodecyl sulfate-polyacrylamide gel electrophoresis (SDS-PAGE) image (Fig. 1a). The His tag was used to facilitate purification of the enzymes, whereas the small SUMO protein fusion (15 kDa) was used as a solubility tag.^38^ The His-SUMO tag was cleaved off with the ubiquitin-like-specific protease 1 (ULP1), resulting in bands of approximately 55 kDa.

The activities of the HALs expressed from different organisms were compared by assessing their abilities to turnover histidine to *trans*-urocanic acid in crude lysates. This was accomplished by monitoring the increase of the absorbance of the product at *λ*_ab_ = 277 nm.^39^ *Alicyclobacillus acidocaldarius* HAL, *Kosmotoga olearia* HAL, *Picrophilus torridus* HAL, and *Thermoplasma acidophilum* HAL all showed some level of histidine conversion into *trans*-urocanic acid (Fig. 1b). *Thermoplasma acidophilum* HAL had the highest level of histidine conversion, even though the concentration in its soluble fraction was rather low. Fusion proteins from lysates that showed substantial abilities to convert histidine to *trans*-urocanic acid, namely *Kosmotoga olearia* His-SUMO-HAL, *Picrophilus torridus* His-SUMO-HAL, and *Thermoplasma acidophilum* His-SUMO-HAL, were purified, and their specific activities in converting histidine into *trans*-urocanic acid were compared. *Thermoplasma acidophilum* His-SUMO-HAL had the highest specific activity, ~30 fold better than *Kosmotoga olearia* HAL and ~3 fold better than *Picrophilus torridus* His-SUMO-HAL (ESI,† Fig. S1a and b). Consequently, *Thermoplasma acidophilum* HAL (TaHAL) was selected for further analysis.

Kinetic studies were conducted at room temperature (23 °C, RT), 37 °C, and 60 °C using purified TaHAL (Table 1). All activity measurements were performed at a pH of 7.4 in order to investigate the possible applications of the enzyme at near neutral pH conditions.

**Fig. 2:**
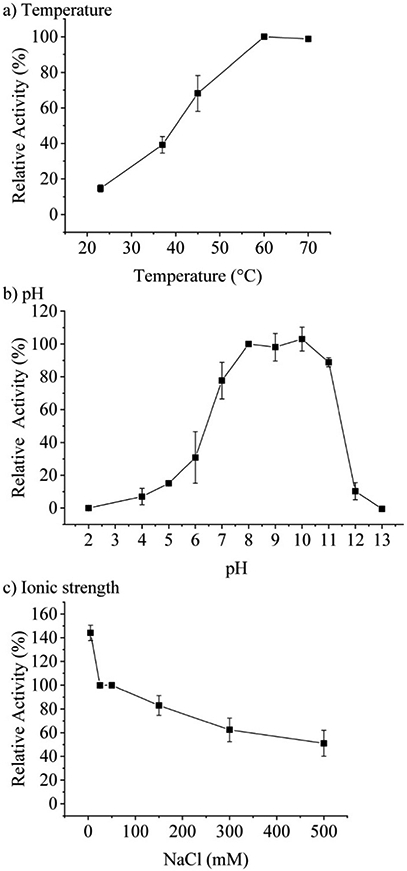
Activity profiles of TaHAL. Temperature (a) and pH (b) profiles of TaHAL (0.1 μM) for the conversion of histidine (0.5 mM) within 30 min expressed as relative activity, normalized to the activity at 60 °C and pH 8, respectively (*n* = 3). (c) NaCl-dependent activity of TaHAL (0.1 μM) at pH 7. The data are normalized to the TaHAL activity at 50 mM NaCl (*n* = 3).

Concentration-dependent measurements of the increase of absorbance at 277 nm over time pointed towards an optimum assay concentration of 0.1 μM of TaHAL (ESI,† Fig. S1c). Therefore, most activity assays of TaHAL in solution were conducted with 0.1 μM of the enzyme. Measuring the temperature-dependent conversion of histidine to *trans*-urocanic acid by TaHAL in buffer A showed an increase in enzymatic activity with a maximal *k*_cat_ at 60 °C (Fig. 2a). In order to assess the stability of the enzyme to temperature stress, we subjected the protein to a circular dichroism (CD) melting experiment (ESI,† Fig. S2a and b). Upon heating the cuvette to 90 °C, only small changes in the absorbance at 222 nm and the spectrum as a whole were observed, indicating that the TaHAL protein retained much of its secondary structure even at high temperatures. The pH profile of TaHAL activity (Fig. 2b) showed an optimum pH between pH 8 and 10. The ionic strength dependent activity of TaHAL shows the enzyme to be active over a broad range of ionic strength conditions (Fig. 2c).

**Fig. 3:**
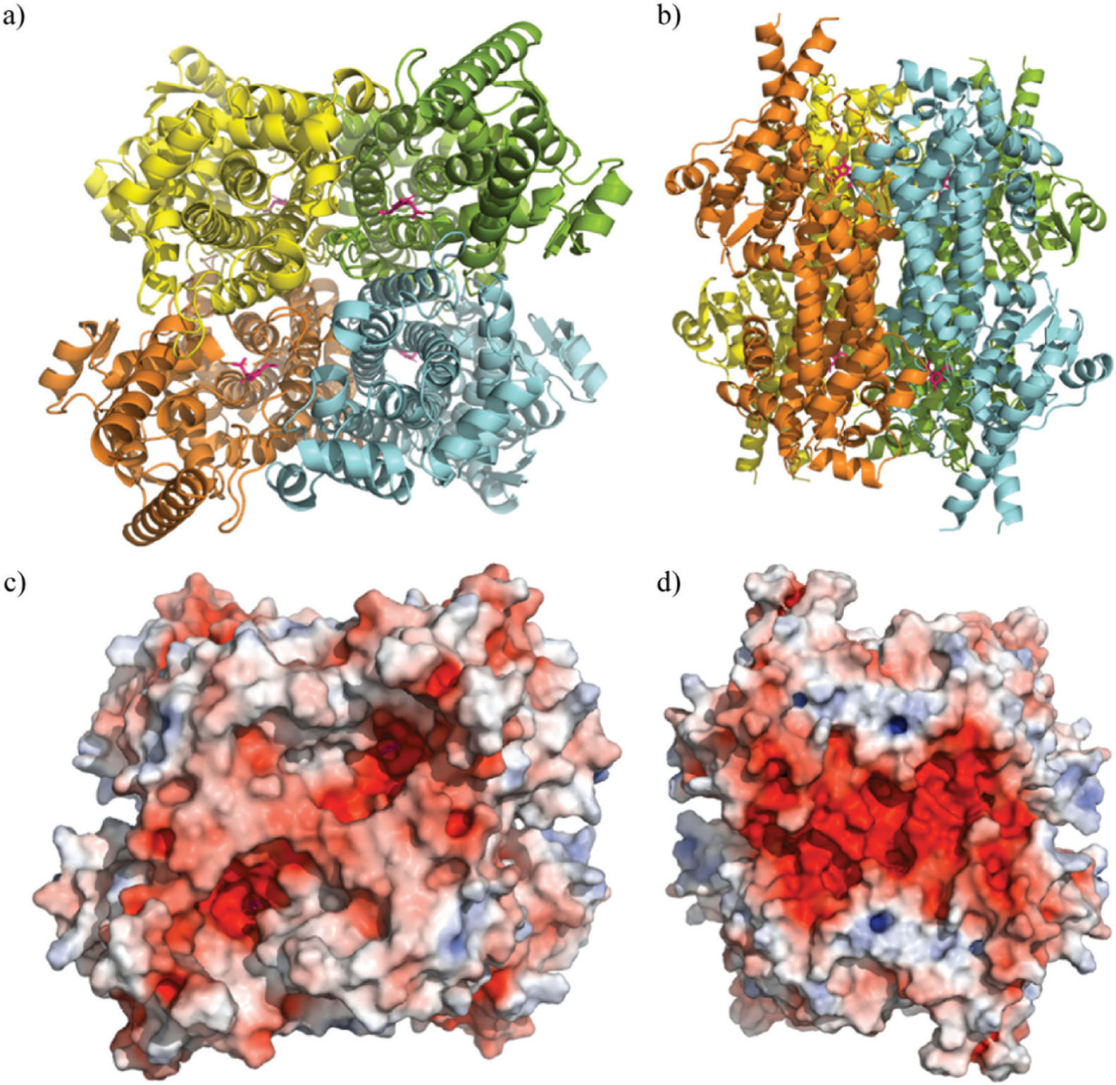
Crystal structure of TaHAL tetramer. Cartoon representations of TaHAL’s structure from a top-down (a) and side-on (b) view. Each unit of the TaHAL tetramer is represented by a different color (yellow, green, orange, cyan), and the MIO catalytic moiety is represented in pink. Surface representations of charge distribution on TaHAL as generated by APBS Electrostatics^7^ in the same top-down (c) and side-on (d) views. Positive charge is represented in blue, hydrophobic patches in grey, and negative charge in red.

The crystal structure of TaHAL was solved, and we found that, similar to other members of the ammonia-lyase family, TaHAL crystallized as a homotetramer (Fig. 3a and b).^40,41^ Moreover, it exhibited electron density consistent with the MIO moiety known to provide catalytic activity in this enzyme class.^40^ Notably, there is a clear enrichment of negatively charged residues on the surface of the protein (Fig. 3c and d). It has been observed that an enrichment of negative charges on the surface of a protein can contribute to solubility and acid stability,^13,19,20,42^ and perhaps the observed enrichment of negative surface charges contributed to TaHAL’s resistance to acid and heat stresses.

### Acid tolerance of engineered TaHAL

**Fig. 4:**
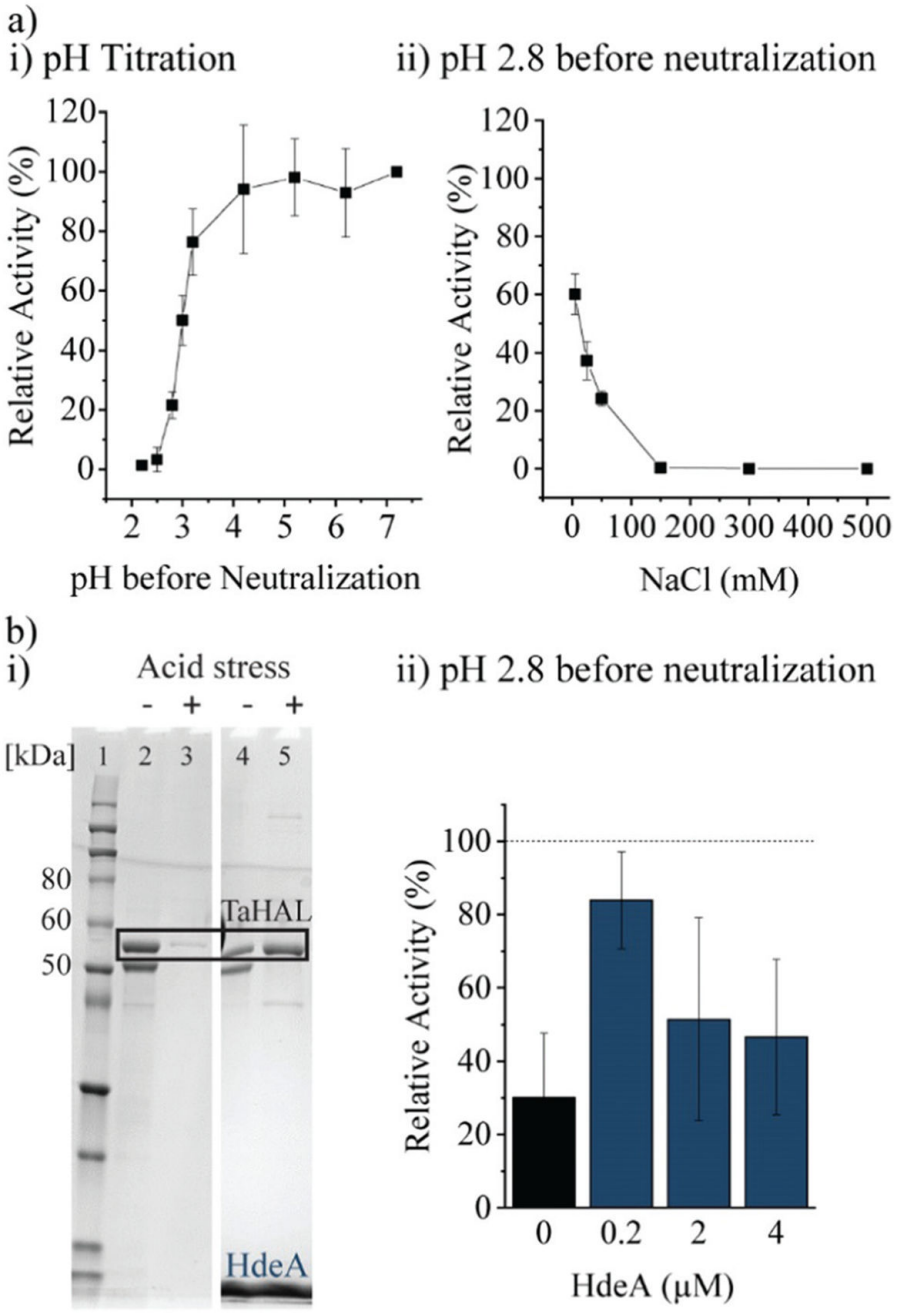
TaHAL acid tolerance. (a-i) Relative activity of TaHAL (0.1 μM) after exposure to different pH environments for 2 h followed by neutralization to pH 7 monitored at 37 °C, 50 mM NaCl. (a-ii) NaCl-dependent activity of TaHAL (0.1 μM) at pH 2.8 (*n* = 3). (b-i) SDS-PAGE of pristine TaHAL (10 μM, lanes 2 and 3), and TaHAL mixed with HdeA (200 μM) (lanes 4 and 5) before and after acidic stress; samples were spun to remove aggregates before loading onto the gel. (Lane 1: Novex™ Sharp Pre-stained Protein Standard.) (b-ii) Relative activity of pristine TaHAL (0.1 μM) and TaHAL mixed with different amounts of HdeA (0.2–4 μM) when incubated at pH 2.8 for 2 h followed by neutralization to pH 7. The data were normalized to the TaHAL activity at pH 7 with the respective HdeA concentration (*n* = 3).

Most important for an enzyme that needs to survive the acidic stomach is its ability to survive acid-mediated denaturation. Since the passage time in the stomach is expected to be relatively short, and since the therapeutic function would be expected in the small intestine where the pH is close to neutral, orally delivered enzymes do not actually need to be active or native at low pH. It is, however, important that an enzyme either does not denature at low pH or more realistically is able to renature following acid-mediated denaturation. In the stomach, the resting pH is at or below pH 2.^43,44^ However, upon eating, the pH will usually rise to be above 5 due to the buffering power of food. The stomach compensates by secreting more acid, although the pH achieved during the fed state can vary widely between individuals between the range of 1 to 6.^43,45^ Minimally, then, in order to withstand the stomach’s pH, a therapeutic enzyme administered with food would need to be resistant to pH ~4, though clearly the more acid resistant the enzyme, the better. The acid tolerance of TaHAL was determined by incubating the enzyme at decreasing pH levels for 2 h at 37 °C to simulate passage through the stomach, followed by pH neutralization to ~7.4 to mimic the transition to the intestine, then followed by activity assessment. We found that TaHAL activity was well maintained with exposure to pH 4, but the activity decreased when TaHAL was incubated at pH 3 and below (Fig. 4a). Exposure to pH 2.8 allowed for the recovery of ~20% activity following neutralization, but 97% of the TaHAL activity was lost following 2 hours at pH 2.5. The pH stability of the enzyme was also investigated using CD spectroscopy (ESI,† Fig. S2c). The native spectrum of the protein indicated mostly alpha-helical content. Upon titration with phosphoric acid, the protein appeared to maintain nearnative secondary structure until a pH of ~3, consistent with the observed partially irreversible loss of activity seen at pH levels below 4.

Next, various additives were tested for their abilities to improve the acid resistance of TaHAL. Arginine, sucrose, and different molecular weights of PEG were tested as additives since they are known to assist in protein refolding.^46-48^ In addition, bovine serum albumin (BSA) and polyphosphate (PolyP) were used due to their described abilities to prevent protein aggregation^49^ and to exhibit chaperone function, respectively, both *in vivo* and *in vitro*.^50^ However, none of these additives had beneficial effects on TaHAL during acid stress (ESI,† Fig. S3a, b and related text).

Additionally, the chaperone HdeA was considered. HdeA is an ATP-independent chaperone that binds to proteins at low pH and then slowly releases them at neutral pH, preventing protein aggregation.^51^ Adding HdeA increased the solubility and activity recovered for TaHAL after acid stress and neutralization (Fig. 4b).

**Fig. 5:**
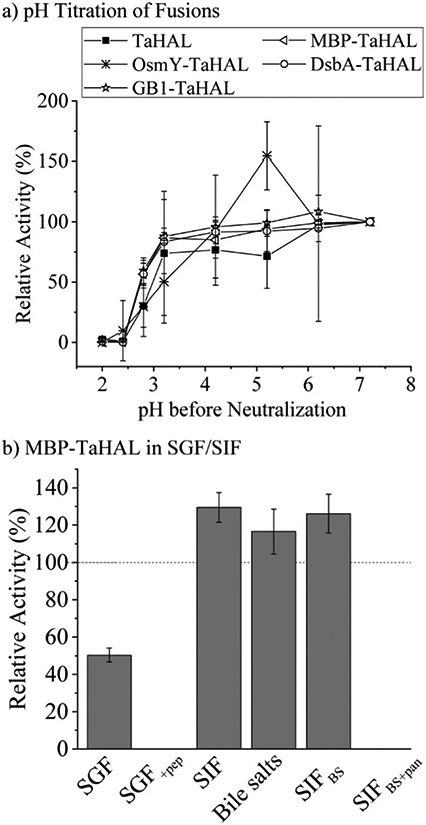
TaHAL fusions and SGF/SIF tolerance. (a) Relative activity of TaHAL and the TaHAL fusions OsmY-, GB1-, MBP-, and DsbA-TaHAL (0.1 μM) after exposure to different pH environments for 2 h followed by neutralization to pH 7 monitored at 37 °C in buffer A (*n* = 3). (b) Relative activity of MBP-TaHAL after incubation for 2 h at 37 °C with different simulated gastrointestinal fluids and neutralization, compared to the enzyme activity in buffer A at pH 7 (*n* = 3).

As a complementary engineering effort, TaHAL was fused to various solubility and chaperone tags. Four different fusions, namely OsmY, maltose binding protein (MBP), DsbA, and the B1 domain of Streptococcal protein G (GB1) were selected. OsmY is a periplasmic chaperone that is soluble up to 400 mg mL^−1^ and has previously been used as a solubility tag for proteins.^52,53^ The periplasmic MBP has been frequently employed for expression in the cytosol, where it is known to enhance the solubility of model proteins when fused to their N-termini. DsbA, which is of periplasmic origin as well, and GB1 have also been used for these purposes.^54,55^ The fusions of TaHAL with GB1, MBP, and DsbA increased the activity after exposure to pH 2.8 and neutralization from ~20% to above 50% activity recovery, whereas no difference was observed for the OsmY fusion compared to the pristine TaHAL (Fig. 5a). Similar results were obtained when HdeA was added to GB1-TaHAL, MBP-TaHAL, or DsbA-TaHAL; no differences were observed among the fusions, but each fusion exhibited increased activity recovery compared to non-fused TaHAL (ESI,† Fig. S3c). We selected MBP-TaHAL for further experiments because it not only increased the activity after exposure to pH 2.8 but also substantially increased the molecular weight of HAL, a feature that was expected to benefit the encapsulation efforts since larger molecules are typically easier to retain.

MBP-TaHAL was then incubated with simulated gastrointestinal fluids to assess the resistance of this fused version of the HAL enzyme to the conditions present in the digestive tract. The gastrointestinal fluids were prepared following the protocol of Minekus *et al*.^45^ Simulated gastric fluid (SGF) consisted of mostly Na^+^ and Cl^−^ ions with a total ionic strength of 90 mM at pH 3. Simulated intestinal fluid (SIF) was made of mostly Na^+^ and HCO_3_^−^ with a total ionic strength of 137 mM at pH 7. Bile salts are natural emulsifiers that can affect orally administrated formulations and active compounds and therefore were added to SIF, resulting in SIF_BS_. In addition to the different salts, the digestive enzymes pepsin and pancreatin were added to SGF and SIF_BS_, respectively, resulting in SGF_+pep_ and SIF_BS+pan_. MBP-TaHAL was incubated with these different gastrointestinal fluids for 2 h at 37 °C before measuring the remaining HAL activity (Fig. 5b). Given the low pH (pH 3) of these incubation conditions and the general sensitivity of proteins to acid-mediated denaturation, it was gratifying to observe that about half the activity was present after incubation in SGF, an observation we attribute to the overall acid resistance of the TaHAL protein. Bile salts did not affect the activity significantly, but the enzymatic activity was obliterated by the addition of digestive proteases to either the low pH or neutral pH buffers.

### Encapsulation of MBP-TaHAL in hydrogels

Given the exquisite protease sensitivity exhibited by MBP-TaHAL, we explored different encapsulation methods. Trapping enzymes in nano-sized vesicles before their encapsulation into larger carrier particles is an approach that can preserve the aqueous 3D environment in the vesicular void and may offer protection against enzymatic degradation by external compounds *e.g.*, proteases. Unfortunately, our efforts to create MBP-TaHAL vesicles led to a decrease in enzymatic activity, possibly due to enzymes becoming associated with the membrane bilayers rather than entrapped within the vesicles (for more details see ESI,† Fig. S4 and S5 and the related text), making subcompartmentalization a non-viable solution in this case.

#### Hydrogel types.

Microreactors can also be assembled *via* direct encapsulation of enzymes into hydrogels. Although these gels lack the hierarchical structure of the vesicular sub-units, their polymer network can nonetheless offer a diffusion barrier within which to trap enzymes, protecting them from environmental proteases. Appropriately constructed hydrogels can resemble the enzymes’ natural environment as a molecularly crowded confined space.^56^ Enzyme-containing hydrogels are used in a variety of applications including tissue engineering, health monitoring, or therapeutic research.^57,58^

**Fig. 6:**
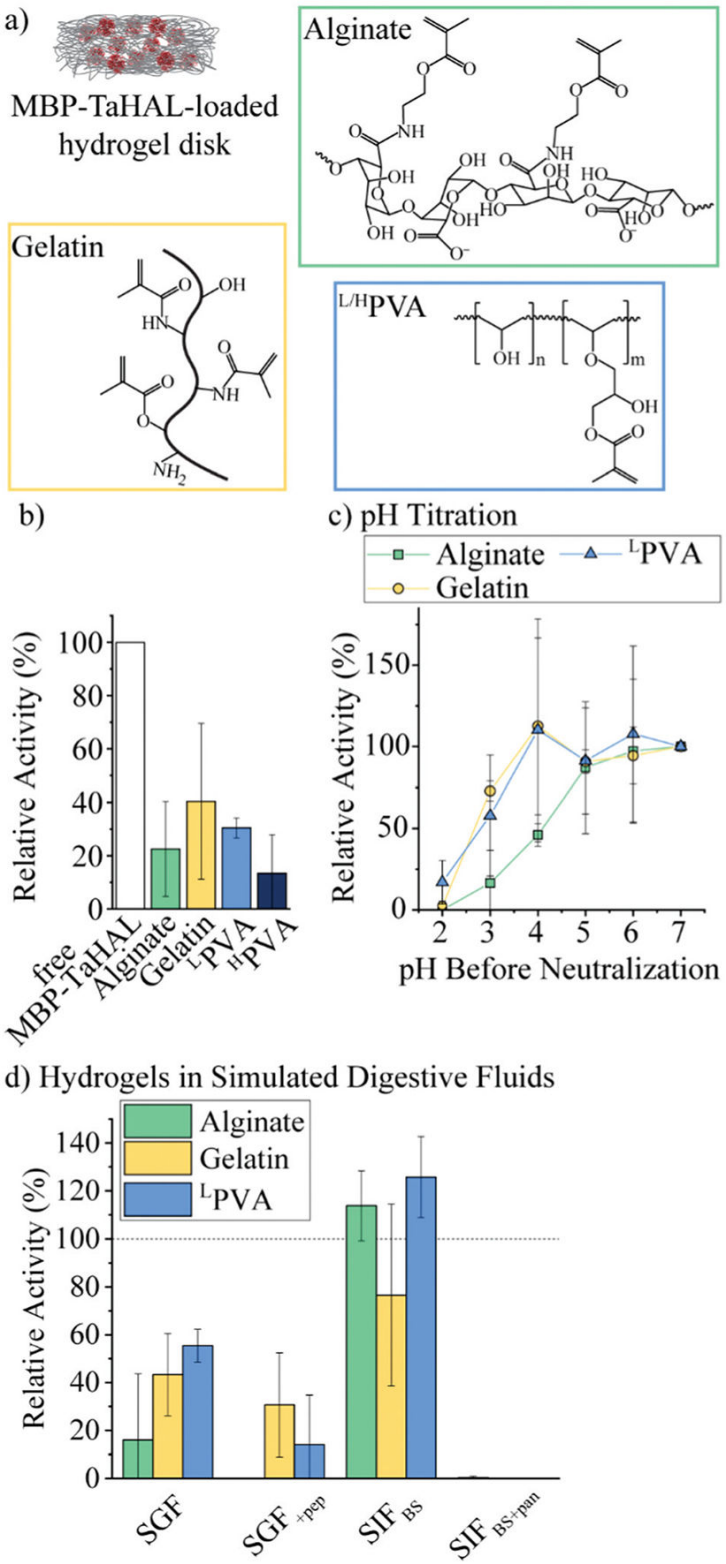
MBP-TaHAL embedded in hydrogel disks. (a) Chemical structures of the polymers used for MBP-TaHAL encapsulation before crosslinking. (b) Relative activity of MBP-TaHAL in solution (1 μM) compared to MBP-TaHAL (1 μM) in alginate, gelatin, ^L^PVA, or ^H^PVA disks. The activity was normalized to the activity of the enzymes in solution (*n* = 2–9). (c) Relative activity of MBP-TaHAL (1 μM) in hydrogel disks after exposure to different pH levels for 2 h at 37 °C followed by neutralization to pH 7. The activities were normalized to the activity in the respective hydrogel at pH 7 (*n* = 3–4). (d) Relative activity of MBP-TaHAL (1 μM) in hydrogel disks after incubation for 2 h at 37 °C with SGF, SGF_+pep_, SIF_BS_, or SIF_BS+pan_, normalized to the activity in the respective hydrogel at pH 7 in buffer A (*n* = 3).

We selected alginate, gelatin, and polyvinyl alcohol (PVA) as three commonly used polymers for biomedical applications that can be photocrosslinked to form very stable hydrogels (Fig. 6a). Alginate is a popular anionic polysaccharide obtained from brown seaweed with diverse applications in food science and biomedicine.^59,60^ Alginate can form hydrogen bonds with the mucin in the intestinal mucus, which could support mucoadhesion of the microreactors.^61,62^ Alginate was modified with an estimated 60% methacrylate groups. This estimate was based on the ratio of the reactants used for the functionalization. It should be noted that it would be difficult to determine the exact modification rate from ^1^H NMR spectra due to overlapping peaks and no clear separation between the peaks of interest. These methacrylate groups allow for the covalent crosslinking of the polymers in the presence of a photoinitiator (*i.e.*, lithium phenyl-2,4,6-trimethylbenzoylphosphinate [LAP]) and upon UV-irradiation. Gelatin-based hydrogels are derived from collagen and have been employed in a variety of biomedical applications.^63^ Gelatin was modified with ~60% methacrylate groups.^64^ Alternatively, PVA is a FDA-approved polymer utilized to make synthetic hydrogels that are widely used for biomedical applications.^65^ Low molecular-weight PVA (^L^PVA) and high molecular-weight PVA (^H^PVA) can be used to construct hydrogels with different degrees of stiffness. PVA was only modified with ~7% methacrylate groups as determined from ^1^H-NMR spectra (ESI,† Fig. S6a). The extent to which PVA was modified was limited because, as other studies have reported, the polymer becomes insoluble in aqueous solutions at higher methacrylate contents.^66^

We aimed to identify the most suitable hydrogel environment that allows for loading, retention, and protection against digestive enzymes of encapsulated MBP-TaHAL. To this end, 50 μL of polymer in buffer A containing 0.1% LAP and 1 μM MBP-TaHAL was placed in a 24-well plate and exposed to UV-light for 30 s, resulting in a crosslinked hydrogel disk with embedded enzymes. Then, 1 mL buffer A was added to the disk in the wells, which were incubated for ~1 min and pipetted up and down, resulting in a washing solution. This washing solution and the hydrogel disks were transferred to a 96-well plate, and 100 μL buffer A containing substrate (0.5 mM histidine) was added. The MBP-TaHAL activity was monitored in a multiplate reader for 2 h (Fig. 6b). In the various hydrogels tested, there was no activity detectable in the washing solution, which indicated that the concentration of enzyme that leaked out of the hydrogel disks in 1 mL washing solution was below the range that exhibited a measurable absorbance signal for the product over the 2 h measurement time. Although there were no statistically significant differences among the MBP-TaHAL activities in the different hydrogels, gelatin had the average highest enzyme activity. ^H^PVA showed the lowest average activity and thus was excluded from further consideration to simplify the analysis.

Next, the pH-dependent change in the enzymatic activity of MBP-TaHAL among the three hydrogels was compared by exposing the enzyme-loaded hydrogel disks to different pH levels at 37 °C for 2 h followed by neutralization to pH 7 and assessment of the catalytic conversion of histidine to *trans*-urocanic acid (Fig. 6c). All values were normalized to the activity of the enzymes in the respective hydrogel disks at pH 7. MBP-TaHAL trapped in gelatin or ^L^PVA disks retained 60–70% activity after incubation at pH 3 for 2 h. However, incubating MBP-TaHAL at pH 2 resulted in a complete loss of activity when it was loaded in gelatin, but residual activity (~20%) remained when it was loaded in ^L^PVA. In contrast, MBP-TaHAL trapped in alginate only preserved ~50%, ~20%, and 0% activity after incubation at pH 4, pH 3, and pH 2, respectively, for 2 h.

Acidic pH is not the only important component of the gastrointestinal milieu; different ions and digestive enzymes are also present. In order to more closely imitate exposure to these stresses, the enzyme-loaded hydrogel disks were exposed to SGF and SIF at 37 °C for 2 h, followed by neutralization to pH 7, and assessment of the catalytic conversion of histidine (Fig. 6d). All data were normalized to the activity of MBP-TaHAL embedded in the respective hydrogel disks at pH 7. As expected, the MBP-TaHAL activity after the exposure to SGF resembled the activity after incubation at pH 3, illustrating that the presence of ~90 mM total ionic strength had no major effect. However, the MBP-TaHAL activity was lowered in all cases when subjected to SGF_+pep_. MBP-TaHAL in alginate disks did not retain any activity, suggesting that, unlike gelatin and ^L^PVA, alginate did not offer protection against proteolytic degradation. The incubation of the MBP-TaHAL-containing hydrogel disks in SIF_BS_ did not affect the enzymatic activity, as previously observed for MBP-TaHAL in solution. However, subjection to SIF_BS+pan_ led to complete losses of MBP-TaHAL activity in all cases, indicating an efficient diffusion of pancreatin through the hydrogel network accompanied by proteolytic degradation of MBP-TaHAL. Moreover, the entire gelatin disks were digested within the 2 h incubation time.

Taking into consideration the observations that MBP-TaHAL encapsulated in alginate lost its activity when exposed to SGF_+pep_ and that pancreatin degraded the entire gelatin disk, we considered ^L^PVA to be the most promising hydrogel environment.

#### MBP-TaHAL in ^L^PVA hydrogel disks.

**Fig. 7:**
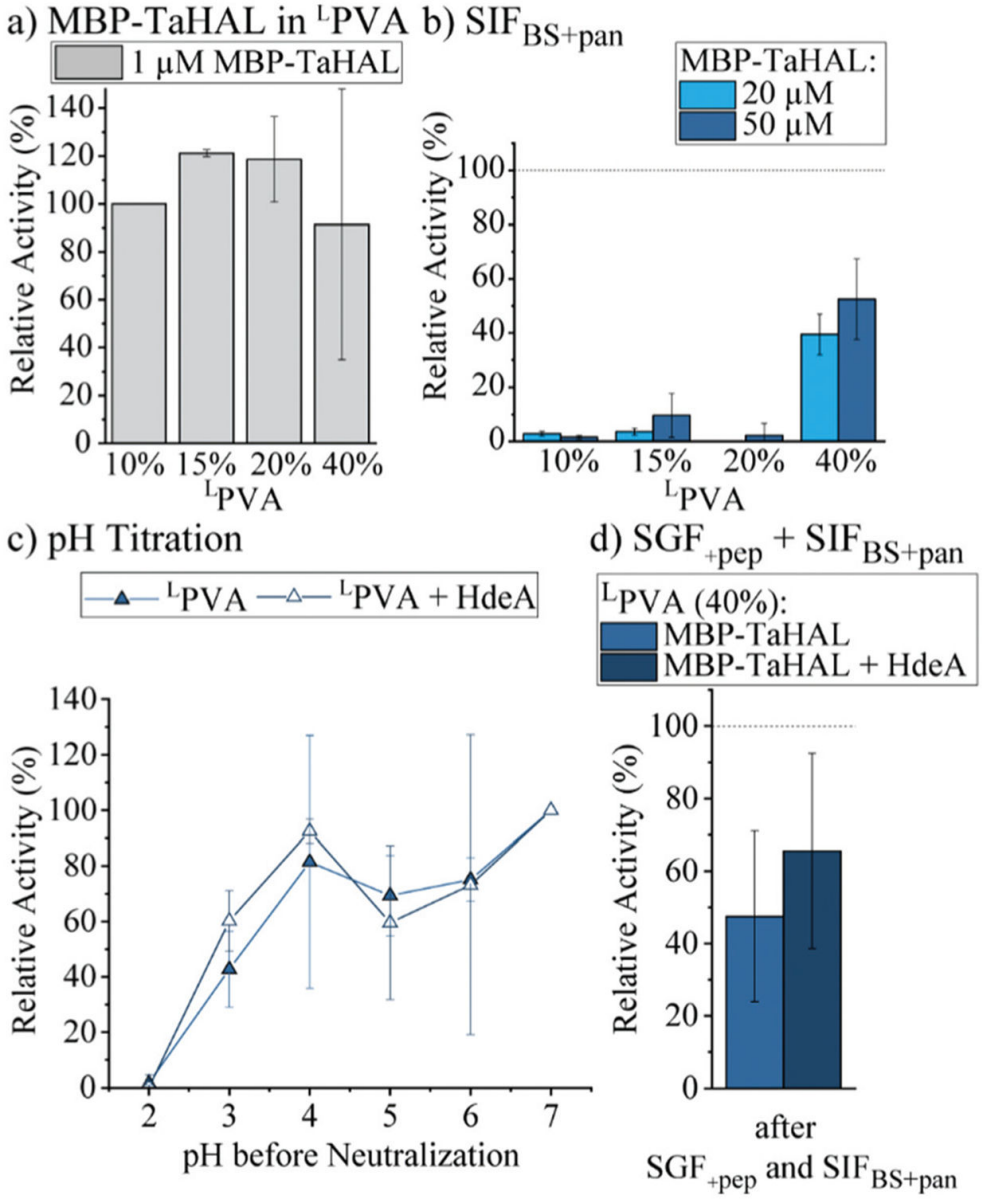
MBP-TaHAL embedded in ^L^PVA disks. (a) Relative activity of MBP-TaHAL (1 μM) in disks made of ^L^PVA (10, 15, 20, and 40% [w/v]). The data were normalized to the activity of MBP-TaHAL in 10% ^L^PVA (*n* = 2). (b) Relative activity of MBP-TaHAL (20 or 50 μM) in hydrogel disks (^L^PVA: 10, 15, 20, or 40% [w/v]) after exposure to SIF_BS+pan_ for 2 h at 37 °C. The data were normalized to the MBP-TaHAL activity after incubation in HEPES buffer at pH 7 (*n* = 3). (c) Relative activity of MBP-TaHAL (50 μM) or MBP-TaHAL and HdeA (50 and 100 μM, respectively) in 40% ^L^PVA hydrogel disks after exposure to decreasing pH for 2 h followed by neutralization to pH 7. The data were normalized to the activity in the respective MBP-TaHAL-loaded disks at pH 7 (*n* = 2). (d) Relative activity of MBP-TaHAL (50 μM) or MBP-TaHAL and HdeA (50 and 100 μM, respectively) in hydrogel disks (^L^PVA, 40% [w/v]) after incubation in SGF with pepsin followed by SIF_BS_ with pancreatin (2 h each), normalized to the activity after an incubation in HEPES buffer at pH 7 (*n* = 3).

Although ^L^PVA was the most promising hydrogel environment for MBP-TaHAL encapsulation, protection from pancreatin clearly needed to be improved. To this end, we increased the density of the ^L^PVA hydrogels to reduce the polymer mesh size and thus exclude pancreatin from the interior of the hydrogel disks. MBP-TaHAL-loaded hydrogel disks were made with increasing concentrations of ^L^PVA up to 40% (w/v). The turnover rate of embedded TaHAL was independent of the ^L^PVA amount, suggesting that ^L^PVA was a benign hydrogel environment (Fig. 7a).

With the aim of improving the catalytic performance of the MBP-TaHAL-loaded ^L^PVA disks, the concentration of the added MBP-TaHAL was increased from 1 μM to 20 and 50 μM. These disks were incubated with HEPES buffer for 2 h, and the activities of the buffer solutions were assessed and used as indicators of enzyme retention within the hydrogel disks (ESI,† Fig. S6b). The reported values were normalized to the activities obtained for the buffer solution incubated with disks made with 50 μM MBP-TaHAL and 10% (w/v) ^L^PVA hydrogels. Increasing the ^L^PVA concentrations resulted in better enzyme retention. The lowest leakage was observed when using 20 μM MBP-TaHAL in 20 or 40% hydrogels. The amount of protein in the buffer solutions of the highest density hydrogel disks containing 1 or 50 μM MBP-TaHAL was quantified with the aim of obtaining an estimate of the MBP-TaHAL mass retained in the hydrogel disks. Approximately 90% of the enzyme remained trapped in the hydrogels when using 40% ^L^PVA disks made with 50 μM MBP-TaHAL, illustrating the very high retention capability of this hydrogel. To test whether product formation exceeded detection limits in experiments with high enzyme concentrations, disks made of 40% ^L^PVA and loaded with MBP-TaHAL (50 μM) were incubated with different substrate concentrations (0.5 and 25 mM) (ESI,† Fig. S6c). The absorbance detection limits were rapidly reached when 25 mM histidine concentrations were used as compared to when 0.5 mM histidine was used. Consequently, an even faster turnover is accomplished with higher concentrations of enzyme embedded in the hydrogels than is measurable by absorbance detection.

Additionally, hydrogel disks made of 10 to 40% ^L^PVA and either 20 or 50 μM MBP-TaHAL were incubated with SIF_BS+pan_ for 2 h at 37 °C before the enzymatic activity was measured (Fig. 7b). Only the disks made of 40% ^L^PVA were able to retain the MBP-TaHAL activity, while hydrogel disks with lower densities (10, 15, and 20%) did not noticeably improve the protection against pancreatin. When 50 μM MBP-TaHAL was encapsulated in 40% ^L^PVA, more than 50% of MBP-TaHAL activity was preserved.

This favorable combination of higher density ^L^PVA (40%) and 50 μM MBP-TaHAL was further evaluated in a pH titration experiment (Fig. 7c). The chaperone HdeA (100 μM) was added for comparison because this chaperone had a beneficial effect on the activity of acid-stressed MBP-TaHAL in solution. As observed for the previously tested conditions, there was a trend towards higher retained activity when incubated at pH 3 in the presence of HdeA. Other than that, there was no difference in the turnover rate of histidine that was dependent on HdeA.

Finally, the activities of ^L^PVA encapsulated MBP-TaHAL after exposure to SGF_+pep_ (2 h, 37 °C) and then SIF_BS+pan_ (2 h, 37 °C) were assessed as a simulation of passage through the stomach and the intestine upon oral administration. The remaining activity was normalized to hydrogel disks incubated in buffer A at 37 °C for 2 h. MBP-TaHAL activity in the order of 50% was preserved, with a trend towards higher activity preservation in the presence of HdeA compared to the activity after an incubation in buffer A (Fig. 7d), illustrating the potential of this formulation for oral delivery of an acid-resistant enzyme.

## Conclusions

We report the identification of a histidine ammonia lyase enzyme that displays significant acid resistance down to pH 2.8. The encapsulation of this enzyme in a high-density photo-crosslinked polyvinyl alcohol hydrogel allowed for the retention of ~50% of its catalytic activity following hours long exposure to simulated digestive fluids including proteases. We conclude that engineered extremophile enzymes in a hydrogel-based formulation present a powerful opportunity to protect biologicals within the gastrointestinal environment.

## Experimental section

### Materials

BamHI-HF, 10-beta competent *E. coli*, BL21 competent *E. coli*, NcoI-HF, Phusion High-Fidelity DNA Polymerase, pMAL-c5X and pMAL-p5X vectors, SHuffle T7 Express Competent *E. coli* (SHuffle B and K12), T4 DNA ligase, and XhoI were purchased from New England Biolabs. LB Agar mix/Lennox was purchased from LabExpress, and 12% Mini-PROTEAN® TGX™ precast protein gel was purchased from Bio-Rad. Isopropyl-β-d-thiogalactopyranoside (IPTG) was obtained from Goldbio. The following products were purchased from Sigma-Aldrich: arginine, complete™, 4-(2-hydroxyethyl)piperazine-1-ethanesulfonic acid (HEPES), ethylene glycol, histidine, imidazole, 2-aminoethyl methacrylate, 4-(dimethylamino)pyridine (DMAP), glycidyl methacrylate (GMA), *N*-(3-dimethylaminopropyl)-*N*′-ethylcarbodiimide hydrochloride (EDC), Sepharose CL-2B, magnesium chloride (MgCl_2_), calcium chloride (CaCl_2_), potassium chloride (KCl), monopotassium phosphate (KH_2_PO_4_), sodium bicarbonate (NaHCO_3_), sodium chloride (NaCl), phosphate buffered saline (PBS), pancreatin from porcine pancreas, pepsin from porcine gastric mucosa, Mowiol® 8–88 (MW 67 000), Mowiol® 4–88 (MW 31 000), lithium phenyl-2,4,6-trimethylbenzoylphosphinate (LAP), polyethylene glycol (PEG)-3350, PEG-8000, PEG-20000, pET22b, phenylmethylsulfonylfluoride (PMSF), sucrose, and tetrahydrofuran (THF). The following products were obtained from Thermo Fisher Scientific: acetic acid (glacial), BODIPY™ 630/650-X NHS Ester (Succinimidyl Ester), Coomassie Brilliant Blue G-250, glycerol, 2-mercaptoethanol (BME), Novex™ Sharp Prestained Protein Standard, PageRuler™ Prestained Protein Ladder, tris(hydroxymethyl)-aminomethane (Tris), and sodium phosphate dibasic (Na_2_HPO_4_). Chloroform anhydrous (≥99%), ethanol, hydrochloric acid (HCl), dialysis tubing with molecular weight cut-off (MWCO) 3.5 kDa (Spectra/por 3), dimethyl sulfoxide (DMSO), membrane filters Nuclepore™ track etched (0.4 and 0.1 μm; Whatman™), *N*-hydroxysuccinimide (NHS) and lysozyme were purchased from VWR. 1-Dipalmitoyl-2-oleoyl-*sn*-glycero-3-phosphocholine (DOPC), 1,2-dioleoyl-*sn*-glycero-3-phosphoethano-lamine (DOPE), cholesterol (chol), and 1,2-dimyristoyl-*sn*-glycero-3-phosphoethanolamine-*N*-(lissamine rhodamine B sulfonyl) (PE-Rho) were purchased from Avanti Polar Lipids. Ni-NTA agarose was obtained from Qiagen. HisTrap HP, Hitrap Q HP, and HiLoad Superdex 200 columns were obtained from GE Healthcare. DNase I was obtained from Invitrogen. dNTP mix was obtained from Promega. Designed primers were ordered from Integrated DNA Technologies (IDT). PRONOVA™ UP LVM sodium alginate and PRONOVA™ UP MVG sodium alginate were purchased from DuPont, USA. The following product was obtained from Hampton Research for crystallography: (±)-2-methyl-2,4-pentanediol. The following products were obtained from Molecular Dimensions for crystallography: strontium chloride hexahydrate, lithium chloride, sodium cacodylate, spermine tetrahydrochloride.

HEPES buffer consisted of 10 mM HEPES and 150 mM NaCl at pH 7.4. Ultrapure water (18.2 M© cm resistivity) was provided by an ELGA Purelab Ultra system (ELGA LabWater, Lane End). Buffer A was prepared as a mixture of 2.5 mM Na_2_HPO_4_ and 50 mM NaCl at pH 7–7.4. Poly(cholesteryl methacrylate)-*block*-poly(2-carboxyethyl acrylate) (BCP1) was synthesized as previously described.^67^

### Cloning

The *E. coli* codon-optimized versions of the *Acidilobus saccharovorans, Caldisphaera lagunensis, Alicyclobacillus acidocaldarius, Picrophilus torridus, Kosmotoga olearia* and *Thermoplasma acidophilum* HAL genes were synthesized and inserted into pUC57 vectors by GenScript. *Acidilobus saccharovorans* HAL and *Caldisphaera lagunensis* HAL were cloned into pET22b, pMAL-p5X, or pMAL-c5X by restriction digestion-mediated cloning. *Alicyclobacillus acidocaldarius* HAL, *Picrophilus torridus* HAL, *Kosmotoga olearia* HAL, and *Thermoplasma acidophilum* HAL were cloned into pET28a containing an N-terminal histidine-SUMO tag for expression. BamHI-HF and NcoI-HF or XhoI from New England Biolabs were utilized as restriction enzymes.

### Protein expression and cell lysis

For protein expression, plasmids (~100 ng) were transformed into *E. coli* BL21 (DE3), SHuffle B, or SHuffle K12, which were plated on LB-agar plates with the appropriate antibiotic (Ampicillin [200 μg mL^−1^] or kanamycin [100 μg mL^−1^]) and incubated at 37 °C for *E. coli* BL21 (DE3) or 30 °C for the SHuffle strains. A single colony was selected and grown in LB media with the respective antibiotic at 37 °C and with shaking overnight. The overnight culture was diluted to an optical density (OD600) of 0.05 in fresh LB media with the appropriate antibiotic and grown at 37 °C and with continuous shaking until an OD600 of 0.8–1 was reached. The culture was then transferred to an incubator at 20 °C 1 h before induction with IPTG (0.1 mM), and overexpression was accomplished by incubation at 20 °C and with shaking for 20 h. For the expression of *Acidilobus saccharovorans* HAL and *Caldisphaera lagunensis* HAL from pET22b, 1 mM IPTG was used for induction, and different expression temperatures (18, 30, and 37 °C) and times (4 h and 20 h) were also tested. Moreover, the expression of *Caldisphaera lagunensis* HAL from pMAL-c5X in SHuffle cells was performed with different induction concentrations of IPTG (0.1, 0.05, and 0.02 mM), and the expression of *Acidilobus saccharovorans* HAL was induced with 0.05 mM IPTG. LB media was exchanged with protein expression media (tryptone [12 g L^−1^], yeast extract [24 g L^−1^], glycerol [50.4 g L^−1^], K_2_HPO_4_ [2.13 g L^−1^], KH_2_PO_4_ [12.54 g L^−1^]) for the expression of TaHis-SUMO-HAL when compared with PtHis-SUMO-HAL.

Lysis of the cells was performed while samples were kept on ice. To conduct lysis, the cultures were spun down and resuspended in lysis buffer (40 mM HEPES, 100 mM NaCl, pH 7.4 or buffer A) supplemented with phenylmethylsulfonylfluoride (PMSF) (1 mM), EDTA-free Protease Inhibitor Cocktail (cOmplete™) (1 tablet per 50 mL), and lysozyme (1 mg mL^−1^). Cells were lysed by sonication (10 × 5 s on, 10 s off, periodicity at 70% power, Fisher Scientific model FB505) followed by centrifugation (16 000*g*, 15–30 min, 4 °C) and separation of the supernatant and pellet. The pellet was resuspended in buffer (40 mM HEPES, 100 mM NaCl) for the SDS-PAGE sample preparation. Supernatant (40 μL) or resuspended pellet were mixed with 10 μL 5 × loading buffer (0.35 M SDS, 6.6 M glycerol, 0.3 M Tris, 0.7 mM bromophenol blue) containing BME (2.5 mM). Samples were boiled at 95 °C for 10 min, and 5 μL of each sample was loaded onto a 12% Mini-PROTEAN gel (BioRad). Five microliters of Novex™ Sharp Pre-stained Protein Standard or PageRuler™ Prestained Protein Ladder were used as molecular weight markers. The gels were run at 150 V for approximately 50 min in a Mini-PROTEAN tetra vertical electrophoresis cell (Bio-Rad) and stained with Coomassie blue stain. Half of each sample for *Acidilobus saccharovorans* HAL and *Caldisphaera lagunensis* HAL expression in the SHuffle strains was mixed with a loading buffer not containing the reducing agent BME in order to check for disulfide bond formation.

### Protein purification

For rapid screening purifications of KoHAL, TaHis-SUMO-HAL, and PtHis-SUMO-HAL, the supernatant of the lysed cells was filtered through a 0.45 μm syringe filter and loaded onto a gravity column with 1 mL Ni-NTA beads per liter expression culture. Samples were washed with 10 mL of buffer (40 mM HEPES, 100 mM NaCl, pH 7.4) and 10 mL wash buffer (50 mM Tris, 100 mM NaCl, 10% glycerol, 100 mM imidazole), followed by elution with either 10 or 1.5 mL elution buffer (50 mM Tris, 100 mM NaCl, 10% glycerol, 500 mM imidazole). Samples were dialyzed overnight against the buffer (40 mM HEPES, 100 mM NaCl, pH 7.4), and, in case of the 10 mL elution fraction, concentrated to approximately 2 mL by spin column (cut off: 50 kDa). Proteins purified with this protocol still contained the histidine-SUMO-tag used for purification purposes. If required, the histidine-SUMO-tag was cleaved using an ubiquitin-like-specific protease 1 (ULP1) upon incubation at 4 °C for 2 h. The concentration of purified protein was estimated *via* absorption measurements at *λ*_ab_ = 280 nm, and an estimated extinction coefficient by the ProtParam tool (ExPASy, Bioinformatics Resource Portal) was used.

For high purity purifications, TaHAL, HdeA- and MBP-TaHAL were purified using the same protocol. *E. coli* BL21 (DE3) containing pET28 constructs for these genes with the His-SUMO N-terminal fusion were grown in 2 L protein expression media with appropriate antibiotic at 37 °C (as described above), then induced overnight with 0.1 mM IPTG at 20 °C. The next day, cells were pelleted and either immediately lysed or frozen at −20 °C for later purification. Cell pellets were resuspended in 100 mL of lysis buffer (50 mM sodium phosphate pH 8.0, 400 mM NaCl, 15 mM imidazole, 0.5 mM MgCl_2_, 10% glycerol) with EDTA-free Protease Inhibitor Cocktail (cOmpletex™) tablets and 0.25 mg DNase I. Samples were sonicated for 5–8 minutes on ice, then clarified by centrifuging twice at 36 000*g* for 30 minutes. The resulting supernatant was loaded onto a HisTrap HP column equilibrated in lysis buffer. The column was washed with lysis buffer, and the protein eluted in lysis buffer supplemented to 500 mM imidazole. This eluate was dialyzed overnight at 4 °C into 40 mM Tris, 300 mM NaCl, pH 8.0 in the presence of ULP1. The next day the protein was passed back over a HisTrap HP column in order to remove both ULP1 and the cleaved His-SUMO tag. HAL proteins, now in the flow through from the His column, were then loaded onto a HiTrap Q HP column in the dialysis buffer, after which they were eluted using an NaCl gradient in 25 mM Tris pH 8.0. Fractions of the eluate containing HAL proteins were then concentrated and subjected to gel filtration chromatography with a HiLoad Superdex 200 column in 40 mM HEPES pH 7.4, 100 mM NaCl. The purity of the protein was confirmed by loading fractions of the HiLoad Superdex 200 elution onto SDS-PAGE gels and staining with Coomassie blue (ESI,† Fig. S7). Fractions of the purified protein were pooled and concentrated before being snap frozen in liquid nitrogen and stored at −80 °C before use.

### Protein engineering

OsmY-TaHAL was constructed using a two-step PCR reaction (Veriti® 96-Well Thermal Cycler, Thermo Fisher Scientific). The primers were designed using Lasergene SeqBuilder. In the first PCR reaction, the OsmY fragment and the TaHAL gene were amplified from their respective vectors. The PCR products were gel-extracted and used as templates in a second PCR reaction that joined the two fragments by their overlapping sequences, which had been generated through the primers designed for the first PCR reaction. Both PCR reactions were performed with the same protocol by mixing 10 μL of 5 × Phu reaction buffer, 0.5 μL of the respective primers (50 μM), 1 μL dNTP mix (10 mM), 50 ng template DNA, and 0.5 μL Phusion High-Fidelity DNA Polymerase (500 U mL^−1^) with ddH_2_O for a total volume of 50 μL. The reaction mix was heated to 98 °C for 30 s followed by 35 cycles of 98 °C for 10 s, 60 °C for 30 s and 72 °C for 2 min, after which it was kept at 72 °C for 6 min and then cooled to 10 °C. The product of the second reaction was again purified by gel extraction and confirmed by sequencing before restriction digest and cloning into pET28a containing an N-terminal His-SUMO tag. DsbA-TaHAL, GB1-TaHAL, and MBP-TaHAL were constructed by the High Throughput Protein Lab at the University of Michigan, then expressed and purified by TriAltus Bioscience.

### Enzyme activity in solution

The enzyme activities were estimated as the initial velocities for all samples. Activities of the purified proteins or that of cell lysates were measured after the addition of either 50 μM or 0.5 mM histidine to samples diluted in buffer A (40 mM HEPES, 100 mM; NaCl, pH 7.4) in quartz cuvettes. TaHAL and histidine solutions (final assay concentrations: 0.1 μM and 0.5 mM, respectively) were incubated in buffer A at several pH levels ranging from pH 2 to pH 13 for 10 min prior to activity measurements to obtain pH profiles. Similarly, the temperature profile was obtained by incubating 0.1 μM TaHAL in buffer A at the respective temperature for 10 min prior to the addition of 0.5 mM histidine. For the concentration-dependent activity of TaHAL, the enzyme was diluted in buffer A in concentrations between 0.02 μM and 10 μM prior to the addition of histidine (0.5 mM). The effect of salt on the enzyme catalysis was investigated by incubating 0.1 μM TaHAL at 37 °C for 2 h in buffer A containing increasing concentrations of NaCl (5–500 mM). The absorption spectrum was measured (*λ*_ab_ = 200–450 nm) at 37 °C at 60 s intervals for 20 min using a temperature-controlled spectrophotometer (Shimadzu UV-1900 or NanoDrop 2000c, Thermo Scientific). The difference between the absorbance at *λ*_ab_ = 277 nm in the initial linear increase and the extinction coefficient 18 800 M^−1^ cm^−1^ of the product *trans*-urocanic acid^39^ were used for calculating the turnover rate per second and the specific activity in units per mg of the enzyme. The results were normalized to the specific activity of TaHAL at 60 °C in the temperature profile, to the specific activity of the enzyme at pH 8 in the pH profile, and to the specific activity of the enzyme in buffer containing 50 mM NaCl in the salt profile to obtain relative activities.

### Kinetics assay

0.2 μM/0.04 μM of purified TaHAL in buffer A were incubated at room temperature (RT)/37 °C/60 °C for at least 5 min prior to mixing with a range of concentrations of histidine (20, 100, 200, 1000, 4000, and 10 000 μM), which were likewise incubated at the respective temperatures. The TaHAL and histidine solutions were mixed half-and-half to a final volume of 500 μL in a quartz cuvette, and absorbance at *λ*_ab_ = 277 nm was immediately measured for 2 min. The measurement for each concentration was repeated twice. The reaction kinetics, calculated by dividing the difference in absorbance over an initial velocity period of 10 seconds by the extinction coefficient of the product (*ε* = 18 800 M^−1^ cm^−1^), were plotted against the substrate concentration. The Michaelis–Menten constant (*K*_m_) and the maximum velocity (*V*_max_) were obtained by fitting the curve to a Michaelis–Menten fit using OriginLab®. The turnover number (*k*_cat_) was calculated by dividing *V*_max_ by the enzyme concentration.

### TaHAL crystallization and data collection

Crystals were obtained by mixing purified TaHAL at concentrations of 20 and 10 mg mL^−1^ at 1 : 1 ratios with a reservoir solution containing 50 mM sodium cacodylate pH 7.0, 50 mM lithium chloride, 12 mM spermine tetrahydrochloride, 52 mM strontium chloride, and 30% v/v (±)-2-methyl-2,4-pentanediol, then incubating using the hanging-drop vapor diffusion method at 20 °C. Upon harvesting, crystals were cryoprotected using the above solution with ethylene glycol added to a concentration of 25% before being flash-frozen in liquid nitrogen.

The diffraction data were collected at the Life Sciences Collaborative Access Team (LS-CAT) beamline 21-ID-F. Diffraction intensities were indexed, integrated, and scaled with CCP4i2.^68^ The crystal belonged to space group *P*622 with one subunit of the tetrameric enzyme in the asymmetric unit (*a* = 169.0 Å, *b* = 169.0 Å, 67.8 Å and *α* = *β* = 90°, γ = 120°). The structure of TaHAL tetramer (shown in Fig. 3) was generated by crystallographic symmetry.

### Structural determination and refinement

The structure was solved by molecular replacement using PHENIX MR with the structure of *P. putida* HAL (pdb: 1GK3) as a search model. Multiple rounds of manual model rebuilding and refinement of the structure were carried out in Coot and PHENIX Refine program. Statistics of X-ray structure determination are listed in ESI,† Table S2.

### Acid stress assay

Hydrochloric acid (HCl, 0.2 M, ~50 μL) was added to the purified enzyme sample in buffer A (500 μL) until a pH of 2 was reached, as measured by a pH meter (Orion Star A211, Thermo Fisher Scientific). The sample was then incubated at 37 °C for 2 h before neutralization to a pH of ~7.2 by the addition of Na_2_HPO_4_ (0.5 M, pH 8) (approximately 50 μL to a sample of purified enzyme in 500 μL). Samples were centrifuged at 16 000*g* for 10 min to separate aggregated protein from the soluble fraction before activity measurements. Acid stress of TaHAL (3 μM) in the presence of additives was conducted in the same way, while arginine (54 mM), sucrose (21 μM), or PEG3350, PEG8000, or PEG20000 (5% w/v) was added before decreasing the pH to 2. The resulting activities were normalized to the activity of TaHAL without additives after acid stress. Similarly, TaHAL (0.1 μM) was incubated with different concentrations of HdeA (0.2, 2, or 4 μM) for 2 h in buffer A, at pH 7 or pH 2.8, before neutralization, whereas the activity values were normalized to the activity of the respective concentration of TaHAL and HdeA at pH 7.

### pH titration

TaHAL (0.1 μM) or fusion constructs (OsmY-TaHAL [0.1 μM], GB1-TaHAL [0.14 μM], DsbA-TaHAL [0.18 μM], or MBP-TaHAL [0.15 μM]) and HdeA (4 μM, if applicable) were incubated for 2 h in a pre-adjusted buffer A at the respective pH (between 7.2 and 2.2) before neutralization. The activity measurements for these samples were performed in 96-well plates (Corning® 96 Well Clear Flat Bottom or Greiner UV-Star® 96 well plates or Corning^®^ UV-Transparent Microplates) in a plate reader (Tecan Infinite M200 PRO Microplate Reader or EnSight, PerkinElmer, USA). After the addition of histidine (0.5 mM final concentration), absorbance was measured at 277 nm at intervals of 60 s for 2 h. When relevant, BSA (1 μM in buffer A) and PolyP (0.5 mM in buffer A) were added with the TaHAL solution into the buffer at different pH values. Similarly, TaHAL (0.1 μM) was incubated with different concentrations of HdeA (0.2, 2, and 4 mMμ) for 2 h in buffer A at either pH 7 or 2.8. The activities of the enzymes were determined as outlined above. Normalization was done in comparison with the activity values of the respective enzyme and additives at pH 7.

### Stability of MBP-TaHAL in simulated digestive fluids

Simulated digestive fluids were prepared in ultra-pure water according to a previously published protocol.^45^ Briefly, the simulated gastric fluid electrolyte solution (SGF) consisted of 6.9 mM KCl, 0.9 mM KH_2_PO_4_, 25 mM NaHCO_3_, 47.2 mM NaCl, 0.1 mM MgCl_2_, and 0.15 mM CaCl_2_. The pH of the solution was adjusted to 3 with 1 M HCl. Pepsin (4000 U mL^−1^ final concentration) was added, yielding the SGF_+pep_. The simulated intestinal fluid electrolyte solution (SIF) consisted of 6.8 mM KCl, 0.8 mM KH_2_PO_4_, 85 mM NaHCO_3_, 38.4 mM NaCl, 0.33 mM MgCl_2_, and 0.6 mM CaCl_2_. The pH of the solution was adjusted to 7 with 1 M HCl. Bile salts (10 μM final concentration) and pancreatin (2 mg mL^−1^ final concentration) were added, yielding the SIF_BS_ and SIF_BS+pan_, respectively. MBP-TaHAL (0.1 μM) was incubated in the different simulated gastric-intestinal fluids at 37 °C for 2 h. SGF and SGF_+pep_ samples were neutralized to pH 7.2 with Na_2_HPO_4_ (0.5 M, pH 8) and centrifuged at 16 000g for 10 min prior to activity measurements, which were conducted as described above. The results were normalized to the specific activity of MBP-TaHAL in buffer A at pH 7 to obtain the stability of the enzyme in digestive fluids.

### Photocrosslinkable hydrogels

Gelatin methacryloyl (GelMA) was made as described previously.^64^ Alginate methacryloyl (AlgMA) was made by dissolving high molecular-weight alginate (PRONOVA™ UP LVM, ^H^Alg) or low molecular-weight alginate (PRONOVA™ UP MVG, ^L^Alg) (990 mg) in PBS buffer and transferring the solutions to round-bottom flasks. NHS (759 mg) and EDC (1.9 g) were added, and each solution was stirred for 15–20 min at room temperature. 2-Aminoethyl methacrylate (545 mg) was added, and the reaction was stirred for 24 h at room temperature before dialysis (3500 MWCO) against ultra-pure water for 3–4 days. The product of ^H^AlgMA or ^L^AlgMA was freeze-dried and stored at −18 °C before use. Polyvinyl alcohol methacryloyl (PVAMA) was synthesized by dissolving 5 g PVA (Mowiol, *M*_W_ 31 or 67 kDa) with 2.5 g DMAP in 125 mL DMSO followed by degassing. GMA (800 μL) was added dropwise, and the mixture was stirred in an oxygen-free environment at room temperature for 48 h. Then, HCl (1 N) was added in a 1 : 1 equivalent to DMAP for neutralization. Finally, the product was dialyzed (3500 MWCO) against ultra-pure water for 5 days and lyophilized, resulting in ^L^PVAMA or ^H^PVAMA. The functionalization degree was determined by taking the integration ratio between the area of the vinyl protons from the methacryloyl groups, which appeared at *δ* 5.7 ppm and *δ* 6.15 ppm, and the area of the methine groups (*δ* 4.1–3.5 ppm) on the backbone of PVA. ^1^H-NMR spectra were taken by a Bruker Ascend 400. MestRe-Nova software was used to analyze the spectra.

### Preparation of UV-crosslinkable hydrogel disks

A 1 : 1 mixture of ^H^AlgMA and ^L^AlgMA (15 mg mL^−1^), GelMA (10% w/v), or PVAMA (67 kDa or 31 kDa; 10, 15, 20, or 40% w/v) was dissolved in buffer A and mixed with LPA (0.1% w/v in water) and the required amount of MBP-TaHAL and HdeA (if applicable). These mixtures (50 μL) were pipetted into the wells of a 24-well plate and crosslinked by UV irradiation (Eurolite LED IP FL-50 COB UV, 395 nm, 50 W) for 30 s. The crosslinked disks were washed with 1 mL HEPES buffer, and 100 μL of this HEPES buffer as well as the disks themselves were transferred to a 96-well plate (Corning® UV-Transparent Microplates), where 100 μL HEPES buffer was added followed by the addition of histidine (0.5 or 25 mM final concentration). Observation of the histidine conversion reaction was performed as outlined above. Leakage of the enzyme from the hydrogels was measured by incubating the hydrogel disks at room temperature in HEPES buffer for 2 h, removing the wash buffer, and determining the enzyme activity in this washing solution after 2 h. The pH titration and the measurements of the stabilities of the encapsulated MBP-TaHAL in hydrogels when exposed to simulated gastrointestinal fluids were conducted as previously outlined. Specifically, the disks were incubated with 1 mL of the required solution for 2 h at 37 °C, washed, and transferred to a 96-well plate, and activity was measured.

## Supplementary Material

supplementary material

## Figures and Tables

**Table 1:** Kinetic properties of TaHAL at different temperatures

Temperature	*K*_m_^a^ (mM)	*k*_cat_^b^ (s^−1^)	*k*_cat_/*K*_m_^c^ (mM^−1^ s^−1^)
RT	0.038 ± 0.01	0.16 ± 0.01	4.21 ± 1
37 °C	0.045 ± 0.02	1.01 ± 0.08	22.44 ± 4
60 °C	0.791 ± 0.20	1.70 ± 0.10	2.15 ± 0.5

a Michaelis–Menten constant (*K*_m_).

b Catalytic constant (*k*_cat_)

c Specificity constant (*k*_cat_/*K*_m_).

## Footnotes

† Electronic supplementary information (ESI) available: Overview over growth conditions, activity of HAL from different organisms, CD data, effect of additives, vesicles and MBP-TaHAL, NMR spectra, **s**tatistics of X-ray structure determination, and HAL amino acid sequences. See DOI: 10.1039/d2ma00051b
