# Supplementary material for: Microreactor equipped with naturally acid-resistant histidine ammonia lyase from an extremophile
Source: Mater Adv. Author manuscript; Available in PMC 2022 Oct 12. (PMC9555226; doi:10.1039/d2ma00051b)
Supplement: supplementary material [file NIHMS1792469-supplement-supplementary_material.docx]

Microreactor Equipped with Naturally Acid-Resistant Histidine Ammonia Lyase from an Extremophile

Carina Ade, Thaís F. Marcelino, Mark Dulchavsky, Kevin Wu, James C. A. Bardwell* and Brigitte Städler*

**Table S1.** Optimum growth conditions for selected organisms.

|  | Temperature (⁰C) | pH | Intracellular pH |
| --- | --- | --- | --- |
| *Acidilobus saccharovorans* | 80 - 85 | 3.5 - 4 |  |
| *Caldisphaera lagunensis* | 70 - 75 | 3.5 - 4 |  |
| *Alicyclobacillus acidocaldarius* | 75 | 3 |  |
| *Picrophilus torridus* | 60 | 0.7 | 4.6 |
| *Kosmotoga olearia* | 65 | 6.8 |  |
| *Thermoplasma acidophilum* | 59 | 1 - 2 | 5.5 |


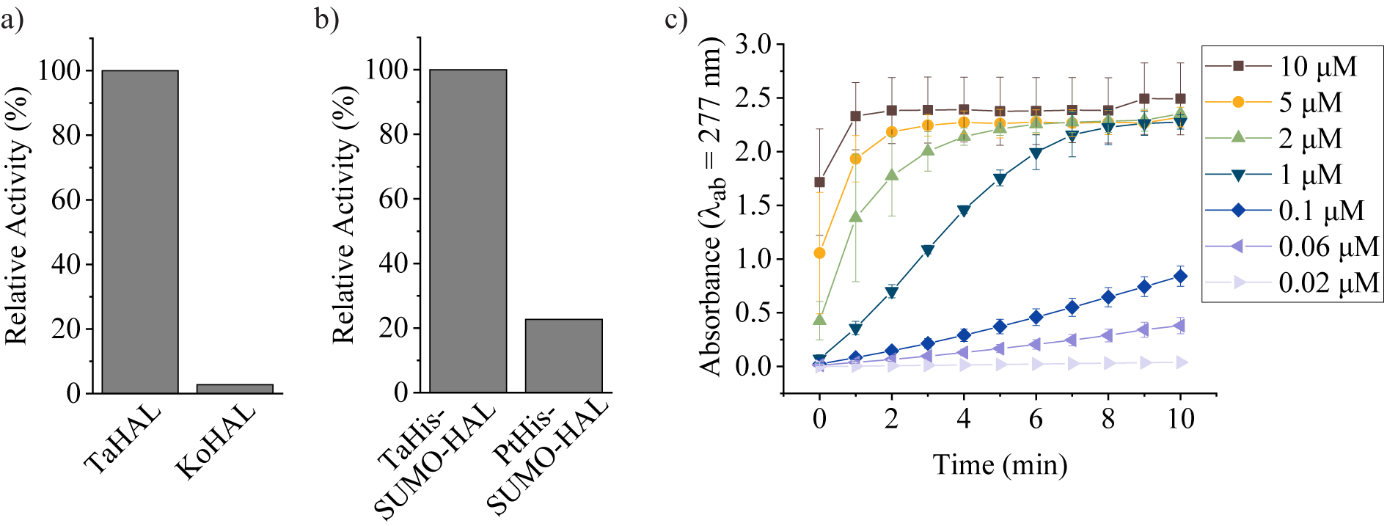


**Figure S1.** a) Relative activity of *Kosmotoga olearia* HAL compared to *Thermoplasma acidophilum* HAL (both 3 μM). b) Relative activity of *Picrophilus torridus* His-SUMO-HAL compared to *Thermoplasma acidophilum* His-SUMO-HAL (both 0.6 μM). c) Time-dependent absorbance curves (λ_ab_ = 277 nm) of *trans*-urocanic acid produced via the conversion of 0.5 mM histidine by TaHAL (0.02 – 10 μM in HEPES buffer) (n = 3).


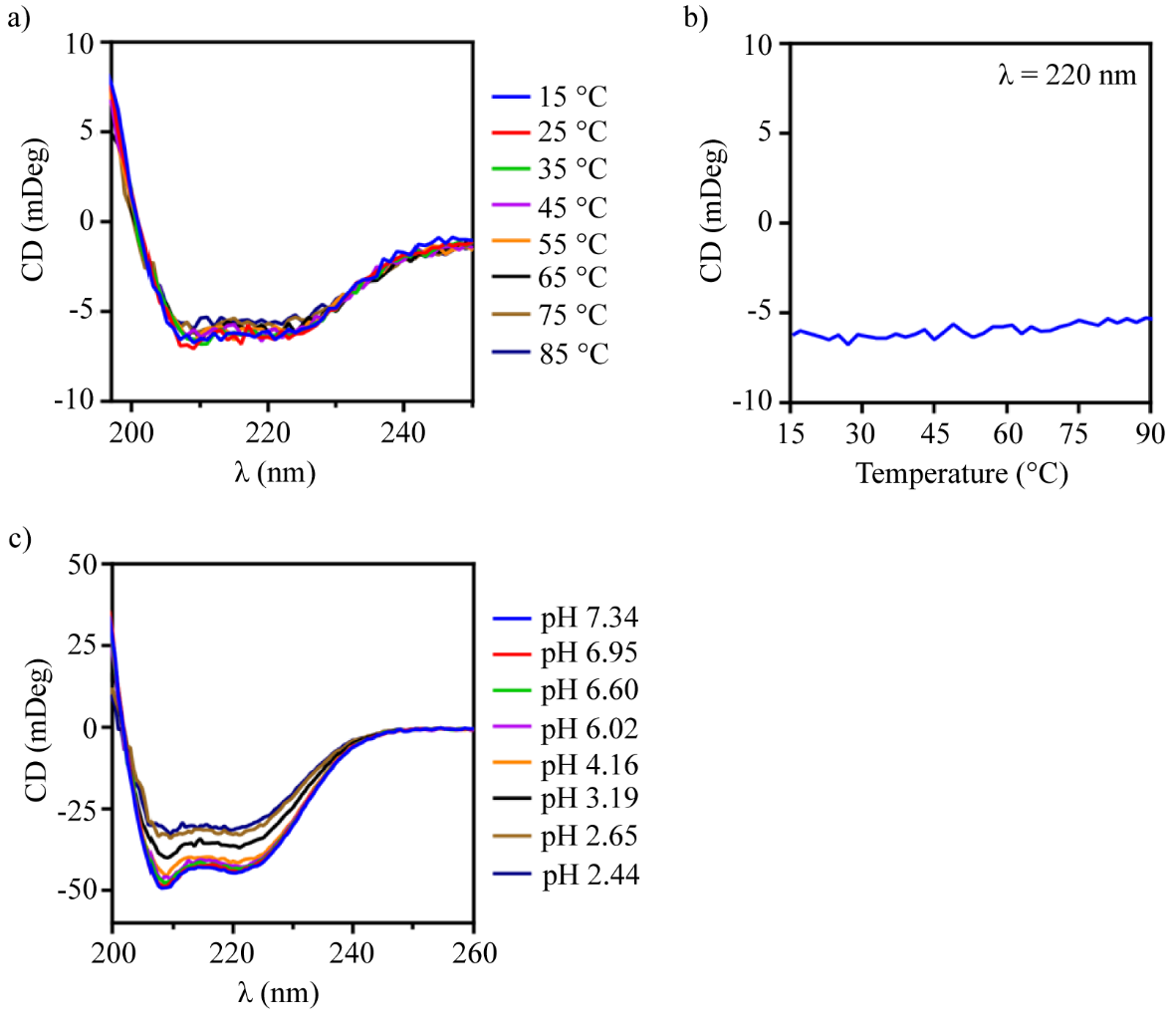


**Figure S2.** Circular dichroism (CD) spectrophotometry of TaHAL. a) CD spectrum of TaHAL (1 µM) obtained in 50 mM sodium phosphate pH 7.0, 50 mM NaCl using a 0.1 cm cuvette in a Jasco J-1500 CD Spectrophotometer. Temperature was ramped from 15 °C to 90 °C at a rate of 2 °C per minute. b) CD signal at 222 nm followed continuously over the course of the melting curve from Figure S2a. c) CD signal of TaHAL was observed over several pH values. TaHAL (1 µM) in buffer (10 mM sodium phosphate, pH 7.4) was monitored in a 1 cm CD cuvette as the solution was titrated using phosphoric acid (1 M) from a pH of ~7.3 to ~2.4. Three spectra were averaged per curve. A loss of signal around the region of 222 nm was apparent, with the greatest magnitude of loss around pH 3.

Effect of Additives on the Acid Tolerance of TaHAL

The ability of BSA to reduce protein aggregation was illustrated by SDS-PAGE (Figure S3ai). Pristine TaHAL (0.2 µM) was compared to the enzyme mixed with BSA (2 μM) and PolyP (1 mM) before (lanes 2 and 4) and after acid stress including neutralization (lanes 3 and 5). In the latter case, a band (lane 5) was observed where the TaHAL was expected, while this band was missing for pristine TaHAL (lane 3). This finding suggests that the additives were beneficial for the solubility of TaHAL after acid stress. The relative activity of pristine TaHAL (0.1 μM) was compared to the activities obtained for TaHAL mixed with BSA (1 μM), PolyP (0.5 mM), or BSA and PolyP when exposed to different pH environments for 2 h followed by neutralization to pH 7 (Figure S3aii). The results showed that TaHAL in the presence of PolyP had a lower tolerance to acid stress i.e., only ~50 % and no TaHAL activity was preserved after exposure to pH 4.2 and 3.2, respectively. On the other hand, pristine TaHAL and TaHAL with BSA showed comparable acid resistance, indicating that the improved enzyme solubility did not translate to higher activity.


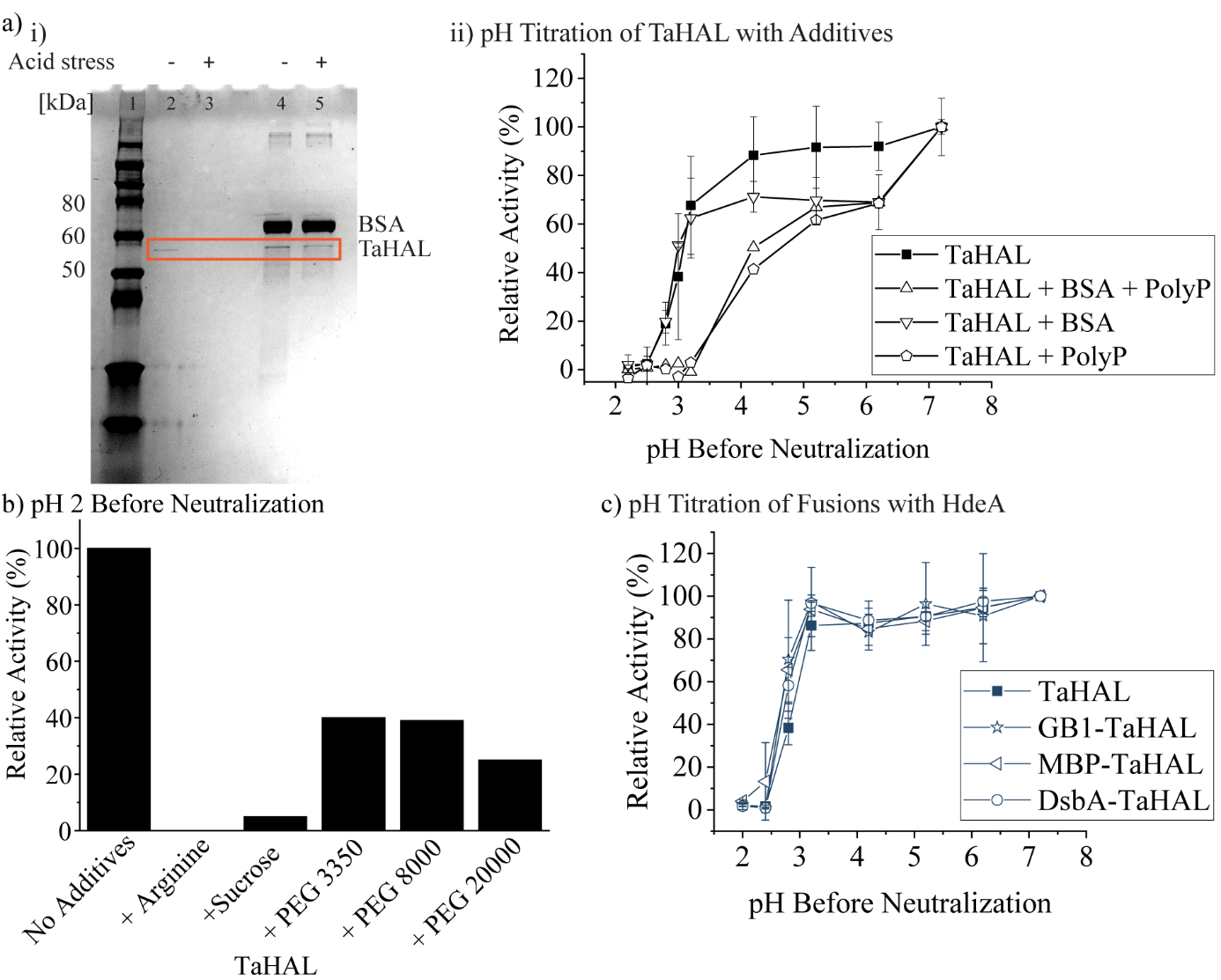


**Figure S3**. Additives in acid stress. ai) Coomassie-stained SDS-PAGE of TaHAL (0.2 μM) alone (lanes 2 and 3) and TaHAL in addition with PolyP (1 mM) and BSA (2 μM) (lanes 4 and 5) before and after acid stress. (Lane 1: Novex™ Sharp Pre-stained Protein Standard). aii) Relative activity of TaHAL alone and in addition with PolyP or BSA after exposure to different pH environments for 2 h followed by neutralization to pH 7 monitored at 37 °C, 50 mM NaCl, and normalized to the activity at pH 7 (n = 1–3). b) Relative activity of pristine TaHAL (3 µM) and TaHAL in the presence of arginine (54 mM), sucrose (21 µM), and different molecular weights of PEG (5 w/v%) after incubation at pH 2 for 2 h and neutralization to pH 7, normalized to the activity after acid stress without additives. (n = 1) c) Relative activity of TaHAL, or the fusions GB1-TaHAL, MBP-TaHAL, or DsbA-TaHAL with HdeA (4 µM) after exposure to different pH values and neutralization, normalized to the respective activity with HdeA (4 µM) at pH 7 (n = 3).

Vesicles and MBP-TaHAL

Experimental Section

Labeling of MBP-TaHAL. BODIPY-NHS Ester (150 µL) was added dropwise to 10 mg mL^-1^ MBP-TaHAL in HEPES buffer. The reaction was allowed to proceed overnight before being followed by dialysis against ddH_2_O for 2 d and freeze-drying to obtain ^f^MBP-TaHAL.

Vesicle Assembly and Interaction with MBP-TaHAL. Hybrid vesicle (HV) assembly was performed according to a previous description.^[1]^

Liposomes were assembled by the same method as the HVs using 1.5 mg (25 mg mL^-1^ in chloroform) DOPC or a combination of 1.41 mg DOPC and 0.099 mg (1 mg mL^-1^ in chloroform) cholesterol, yielding L_PC_ or L_PC, chol_. ^Rho^L_PC_ was assembled by mixing 4.875 mg DOPC and 0.125 mg (1 mg mL^-1^ in chloroform) 1,2-dimyristoyl-sn-glycero-3-phosphoethanolamine-N-(lissamine rhodamine B sulfonyl) (PE-Rho).

^f^MBP-TaHAL (1 µM) was added to the pre-assembled vesicles (L_PC_, L_PC, chol_, HV_PC_, or HV_PE_) and incubated for 15 min at room temperature before the read out of fluorescence intensity (λ_ex_ = 600 nm, λ_em_ = 640 nm). The values were normalized to the fluorescence intensity of free ^f^MBP-TaHAL (1 µM in HEPES buffer).

Additionally, the enzyme was added in the rehydration step in order to determine the association of MBP-TaHAL with the vesicles throughout the formation and purification process. Therefore, vesicle samples (^Rho^L_PC_, L_PC_, L_PC, chol_, HV_PC_, or HV_PE_) were rehydrated with HEPES buffer containing MBP-TaHAL or ^f^MBP-TaHAL (10 µM). The fluorescence intensity (λ_ex_ = 600 nm, λ_em_ = 640 nm) of each fraction with ^f^MBP-TaHAL before (diluted to 10×) and after SEC was measured in a black well plate by a plate reader (EnSight, PerkinElmer, USA, or BioTek, Synergy H1, USA). Similarly, the fluorescence intensity (λ_ex_ = 488 nm, λ_em_ = 596 nm) of each fraction with ^Rho^L_PC_ was recorded. The fluorescence intensities were normalized to the highest value before SEC or to the values of the respective first fractions after SEC. Activity of the enzyme was measured through absorption as outlined above with normalization to the highest values in each sample.

Giant Unilamellar Vesicle (GUV) Assembly and Interaction with MBP-TaHAL.

GUVs were made following a previous protocol.^[1]^ Briefly, GUVs were assembled using either 10 µL DOPC (25 mg mL^-1^ in chloroform) and 0.5 µL RhoPE (1 mg mL-^1^ in cholorform) resulting in ^Rho^GUV_PC_ or 8.36 µL DOPC (25 mg mL^-1^ in chloroform), 0.3 µL cholesterol (25 mg mL^-1^ in chloroform) and 0.5 µL RhoPe (1 mg mL^-1^) resulting in and ^Rho^GUV_PC,chol_. 290 µL sucrose buffer was used for rehydration. The interaction of MBP-TaHAL with ^Rho^GUV_PC_ or ^Rho^GUV_PC,chol_ was imaged with a confocal laser scanning microscope (CLSM, Carl Zeiss, Germany). GUV solution (2–5 μL) was added to a drop of 25 µL HEPES buffer placed on a glass coverslip. Then, 3 μL MBP-TaHAL or ^f^MBP-TaHAL solution (40 µM) was added to this drop. Images of three to five different areas were recorded before and after the addition of MBP-TaHAL.

Results and Discussion

Given the clear protease sensitivity exhibited by MBP-TaHAL, we decided to assemble subcompartmentalized microreactors to protect it by encapsulation. We attempted to encapsulate this enzyme into previously characterized vesicles made of the amphiphilic block copolymer BCP1 and either DOPC (HV_PC_) or DOPE lipids (HV_PE_). These carriers were chosen in part because poly(2-carboxyethyl acrylate) had a hydrophilic-to-hydrophobic phase transition at pH ~4.^[2]^ The collapsed polymer chains were anticipated to offer a protective barrier in the stomach that returns to a hydrophilic state in the intestine and does not hinder substrate diffusion. We previously reported that HV_PC_ retained its structural integrity when cycling from pH 7 to 4 and back to pH 7.^[1]^ Alternatively, to test more standard carriers that do not have these pH responsive characteristics, liposomes were assembled from either DOPC lipids (L_PC_) or from DOPC and molecular cholesterol in the same amounts present in the hydrophobic block of BCP1 (L_PC, chol_). Since cholesterol is known to affect lipid bilayer permeability, it is important to evaluate whether potential differences between L_PC_ and HV_PC_ were due to the presence of cholesterol or due to the BCP1.

The film-rehydration method was used to prepare vesicles from all these different components. MBP-TaHAL was incorporated into these vesicles by adding it to the rehydration buffer. MBP-TaHAL was furthermore labeled with BODIPY® 630/650-X dye (^f^MBP-TaHAL) in order to follow this enzyme during the assembly process. All samples were extruded through a 400-nm membrane followed by size exclusion chromatography (SEC) in order to purify vesicles containing MBP-TaHAL from free enzymes and smaller assemblies.

We first confirmed that the labeled enzyme remained >70% active (Supporting Information Figure S4a). The fluorescence intensities of the collected fractions following extrusion and SEC purification of ^f^MBP-TaHAL were measured (λ_ex/em_= 600/649 nm), and the data were normalized to the fluorescence intensity of the first fraction. The results showed that most of ^f^MBP-TaHAL cofractionated with the vesicles (Supporting Information Figure S4b). (It should be noted that the HV_PE_ concentration was lower than the other vesicle concentrations, likely due to a less successful rehydration step since the vortexing time was limited to preserve the ^f^MBP-TaHAL activity i.e., less ^f^MBP-TaHAL associated with the vesicles and more enzyme eluted in the later fractions.) Additionally, the ^f^MBP-TaHAL activity of each of the fractions for all assemblies was measured (Supporting Information S4c). Surprisingly, there was no ^f^MBP-TaHAL activity detected in the fraction that contained the vesicles (the first fraction). The highest turnover rates were detected for fractions 4 and 5, which corresponded to the enzymes not associated with the vesicles. Experiments performed with unlabeled MBP-TaHAL showed that these observations were not related to the presence of a fluorescence label on MBP-TaHAL (Supporting Information Figure S4d). We determined that enzymatic activity was not significantly affected by exposure to the SEC column (Supporting Information Figure S4e). In contrast, when mixing empty vesicles with MBP-TaHAL, ~50% of the enzymatic activity was lost, suggesting that the presence of the vesicles had a negative effect on the function of MBP-TaHAL.

It is typically assumed/expected that the enzymatic cargo is encapsulated in the void of vesicles due to the hydrophilic nature of the proteins. However, it is also possible for proteins to associate with the membranes of vesicles, which could explain the loss of MBP-TaHAL activity. To test for this association, pre-made vesicles and ^f^MBP-TaHAL were mixed together, and the fluorescence intensity was measured. BODIPY® 630/650-X, the dye used to label MBP-TaHAL, is known to have higher fluorescence intensity in hydrophilic environments, e.g., when incorporated in a lipid membrane. The fluorescence intensity of the vesicle-^f^MBP-TaHAL mixture was ~2× higher than that of pristine ^f^MBP-TaHAL, supporting the conclusion that ^f^MBP-TaHAL was incorporated into the vesicles’ membranes (Supporting Information Figure S4f). As mentioned above, the limited vortexing time during HV_PE_ assembly likely resulted in a lower HV_PE_ concentration, and consequently, the difference between pristine ^f^MBP-TaHAL and a mixture of ^f^MBP-TaHAL and HV_PE_ was smaller.


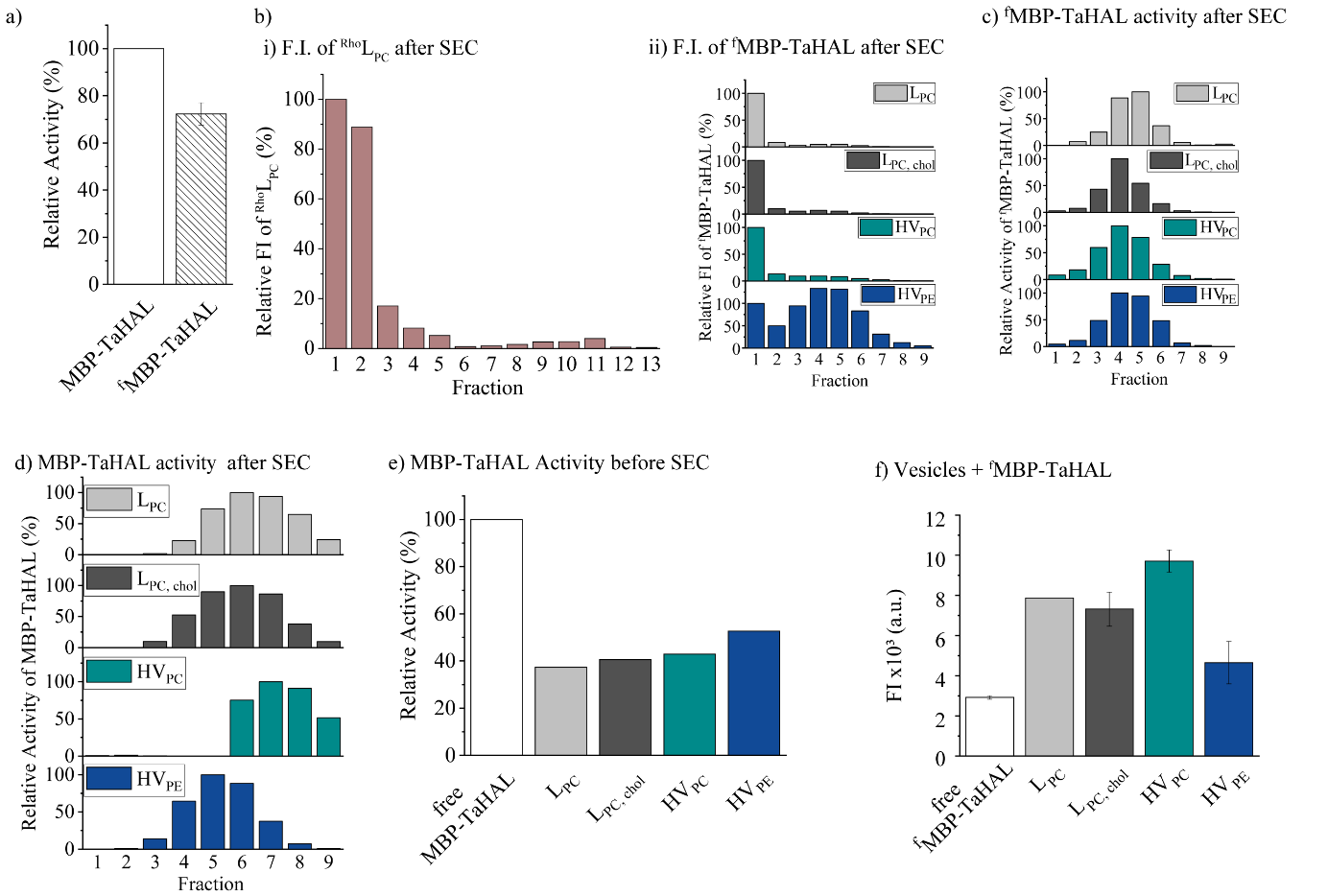


**Figure S4**. MBP-TaHAL associated with nano-sized vesicles. a) Relative activity of MBP-TaHAL compared to ^f^MBP-TaHAL. b) i) Relative fluorescence intensity (FI) (λ_ex_ = 488 nm, λ_em_= 596 nm; normalized to fluorescence intensity at λ_em_= 596 nm of fraction 1) of the fractions after SEC of rhodamine-labeled ^Rho^L_PC_ with MBP-TaHAL (10 µM). ii) Relative fluorescence intensity of ^f^MBP-TaHAL the vesicle-containing fractions (L_PC_, L_PC, chol_, HV_PC_, or HV_PE_) after SEC when ^f^MBP-TaHAL (10 µM) was added to the rehydration buffer. The data were normalized to the fraction with the highest fluorescence intensity/activity. c) Relative activity of ^f^MBP-TaHAL (c) or MBP-TaHAL (d) in the vesicle-containing fractions (L_PC_, L_PC, chol_, HV_PC_, or HV_PE_) after SEC when ^f^MBP-TaHAL (10 µM) was added to the rehydration buffer. The data were normalized to the fraction with the highest fluorescence intensity/activity. e) Relative activity of MBP-TaHAL in solution and MBP-TaHAL in the presence of vesicles (L_PC_, L_PC, chol_, HV_PC_, and HV_PE_) in solution before SEC. The data were normalized to the activity of the enzyme in solution. f) Fluorescence intensity (λ_ex/em_ = 600/640 nm) of pristine ^f^MBP-TaHAL (1 µM) or ^f^MBP-TaHAL (1 µM) mixed with pre-assembled vesicles L_PC_, L_PC, chol_, HV_PC_, or HV_PE_ (n = 1–3).

Giant unilamellar vesicles (GUVs) are a useful model to visualized membrane interaction events using confocal laser scanning microscopy (CLSM). GUVs were made via the electroformation method to image their interaction with ^f^MBP-TaHAL. To this end, GUVs consisting of DOPC lipids or of DOPC lipids with cholesterol and PE-Rho were assembled and referred to as ^Rho^GUV_PC_ and ^Rho^GUV_PC, chol_. It was not possible to fabricate GUVs with incorporated BCP1. CLSM images of the GUVs were taken before and 10 min after the addition of ^f^MBP-TaHAL (left and right image in Supporting Information Figure S5a, respectively). Spherical non-interacting ^Rho^GUV_PC_ and ^Rho^GUV_PC,chol_ were observed before the addition of ^f^MBP-TaHAL. In contrast, the addition of ^f^MBP-TaHAL resulted in an overlap of the fluorescent signal originating from the GUVs’ membranes and ^f^MBP-TaHAL (i.e., GUVs’ membranes appeared violet due to the overlay of the false-red color originating from PE-Rho and the false-blue color originating from ^f^MBP-TaHAL). No fluorescent signal was observed in the void of the GUVs, suggesting that the enzyme could not cross the membrane. In addition, the GUVs closely stuck together resulting in a honeycomb-like structure after the association of ^f^MBP-TaHAL with the membranes of the GUVs. The presence of cholesterol in the GUVs membrane did not detectably change the interaction with ^f^MBP-TaHAL. Similar behavior was observed when using MBP-TaHAL, supporting the assumption that the morphological changes in the GUVs were due to the enzyme becoming incorporated into the membrane and not due to the rather hydrophobic dye BODIPY® 630/650-X dye (Supporting Information Figure S5b). In order to remain active, TaHAL needs to remain in a folded, catalytically competent state. This protein exists as an active tetramer, with structure of the active site dependent on this tetrameric assembly. If association with the vesicle membrane results in unfolding of TaHAL or disassociation of the active tetramer, that would result in the observed loss of activity.

Overall, the MBP-TaHAL inactivation due to the association with the membranes of all tested types of vesicles made the concept of subcompartmentalization impossible.


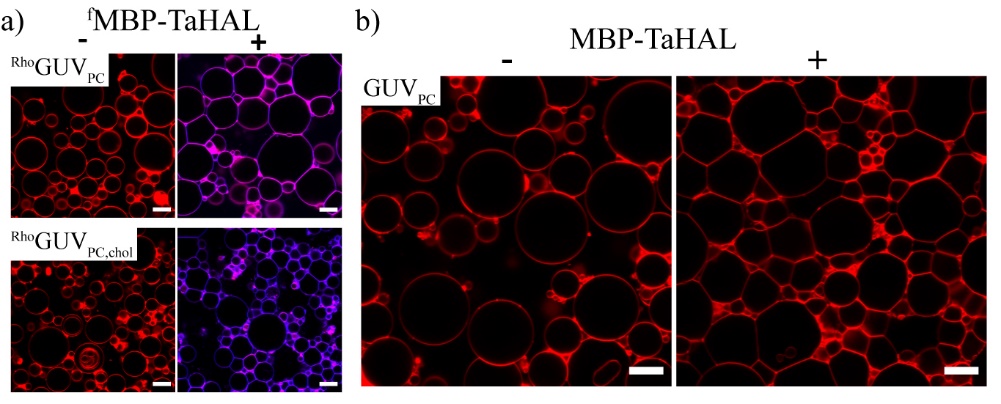


**Figure S5.** a) ^Rho^GUV_PC_ (top images) and ^Rho^GUV_PC, chol_ (bottom images) before the addition of ^f^MBP-TaHAL (left image) and after the addition of ^f^MBP-TaHAL (right image) (scale bars 10 µm). b) ^Rho^GUV_PC_ before (left image) and ^Rho^GUV_PC_ after (right image) the addition of MBP-TaHAL (scale bars 10 µm).


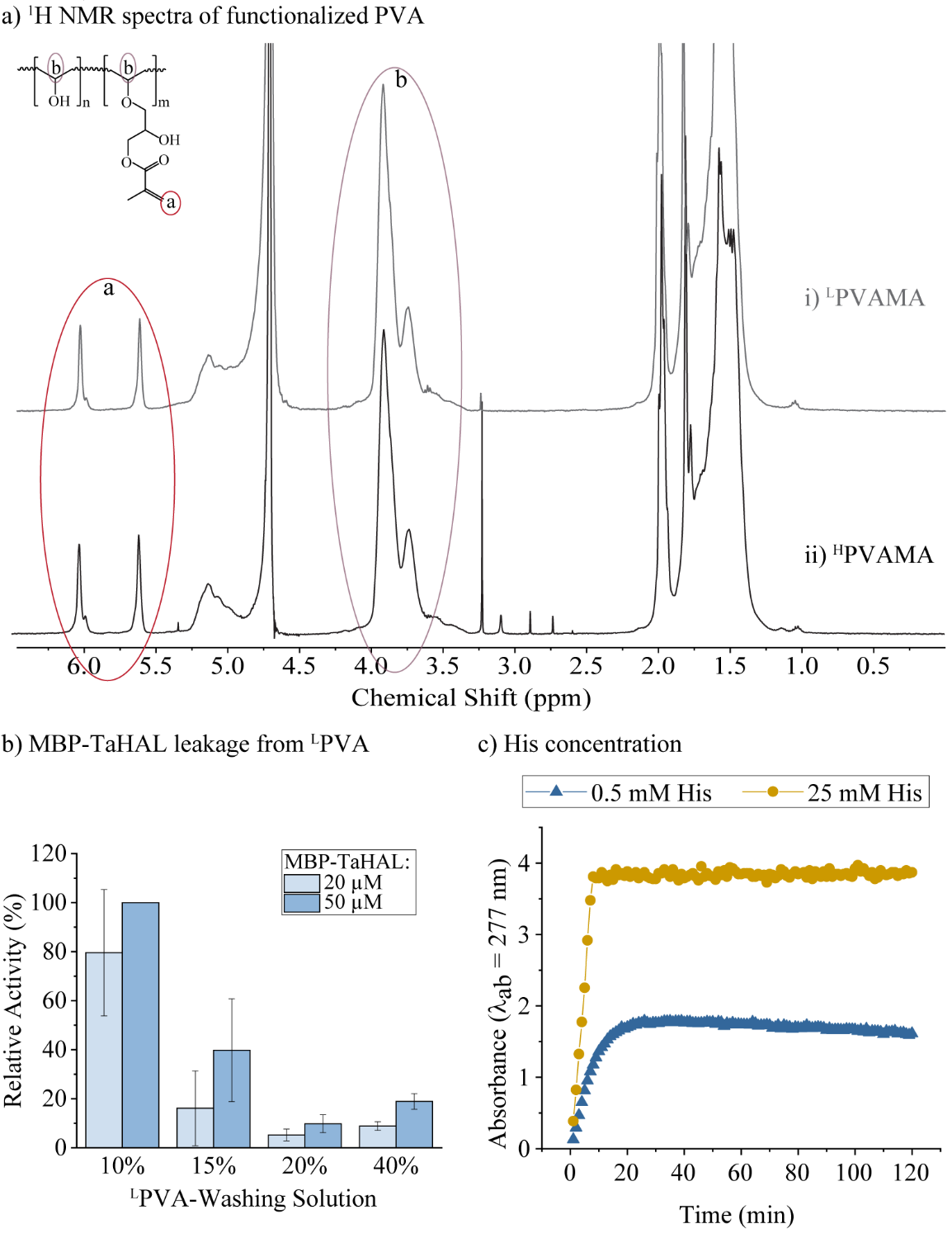


**Figure S6**. ^L/H^PVAMA and activity of MBP-TaHAL in ^L^PVA disks. a) ^1^H NMR spectra of ^L^PVAMA (i) and ^H^PVAMA (ii) in D_2_O (400 MHz). b) Relative activity of the washing solution of ^L^PVA disks (10, 15, 20, or 40% [w/v]) with MBP-TaHAL (20 or 50 µM) after 2 h in HEPES buffer, normalized to the highest remaining activity (n = 3). c) Time-dependent absorbance curves (λ_ab_ = 277 nm) of *trans*-urocanic acid produced via the conversion of 0.5 mM or 25 mM histidine (His) by MBP-TaHAL (50 μM in ^L^PVA disks [40%, w/v] and HEPES buffer).


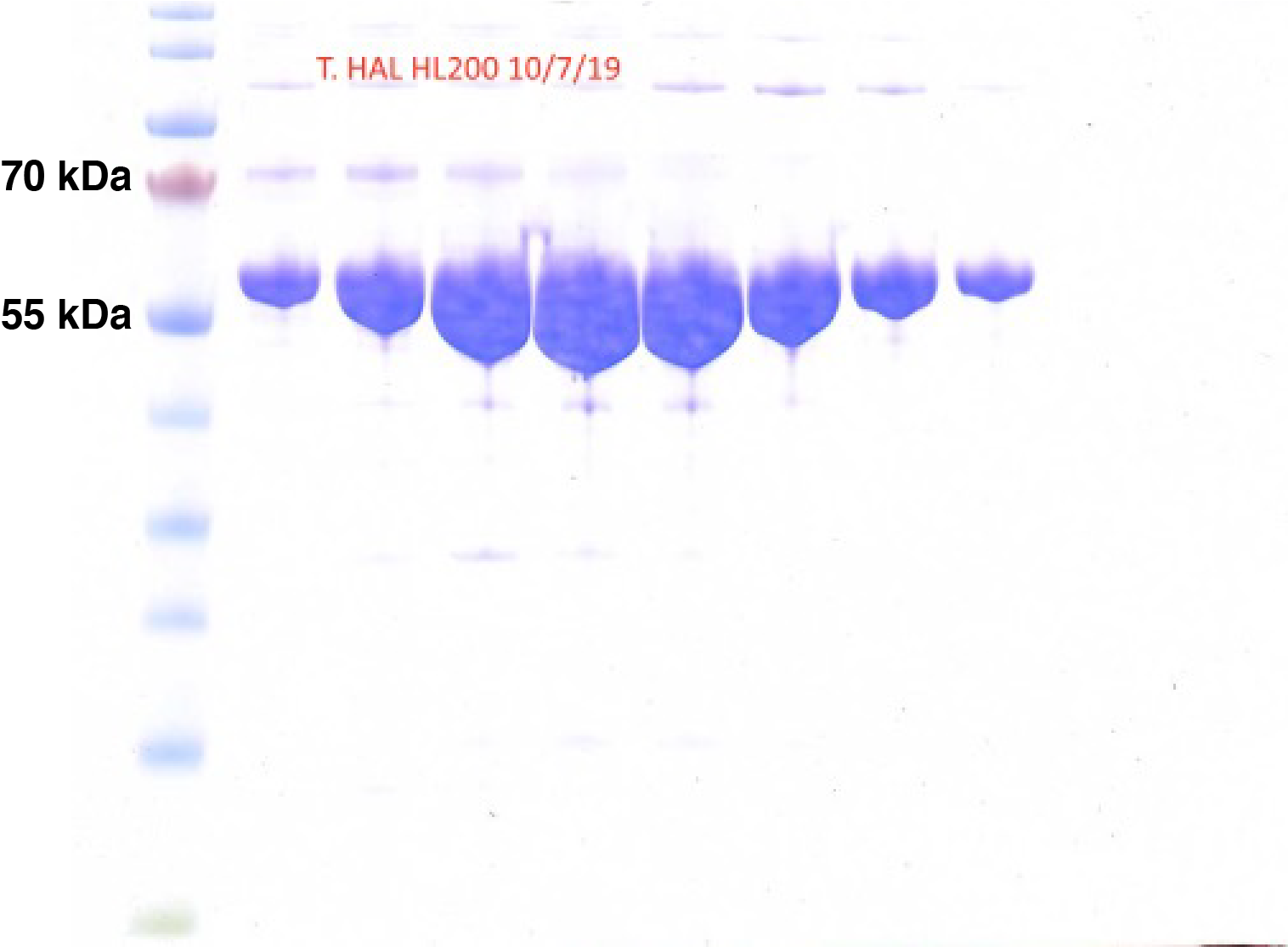


**Figure S7.** Coomassie blue gel of purified TaHAL. TaHAL was purified according to the high purity purification protocol described in the methods sections. Fractions of the HiLoad Superdex 200 elution were loaded onto SDS-PAGE gel and stained with Coomassie blue. All fractions were pooled and snap frozen before being used for in vitro experiments.

**Table S2.** Statistics of X-ray structure determination

| **Data collection** |  |
| --- | --- |
| Space group | P 6 2 2 |
| Cell dimensions |  |
| *a, b, c* (Å) | 169.0, 169.0, 67.8 |
| α, β, γ (°) | 90.0, 90.0, 120.0 |
| Resolution (Å) | 52.86-2.10 |
| R_merge_ (%) | 10.1 (64.6) |
| R_meas_ (%) | 10.3 (66.4) |
| *I/σ(I)* | 19.7 (4.8) |
| CC_1/2_ | 0.999 (0.961) |
| Redundancy | 18.1 (17.9) |
| Completeness (%) | 100 (100) |
| Total/unique reflections | 610086/33715 |
| **Refinement** |  |
| Resolution (Å) | 52.86-2.10 |
| No. unique reflections | 33715 |
| R_work_, R_free_ (%) | 14.6, 17.9 |
| No. atoms | 3817 |
| Protein | 3701 |
| Ligand | 0 |
| Water | 116 |
| Rotamer outliers (%) | 1 |
| Clashscore | 4 |
| RMSD |  |
| Bond lengths (Å) | 0.014 |
| Bond angles (°) | 1.452 |
| Average B factors (Å^2^) | 38.0 |
| Wilson B-factor (Å^2^) | 30.7 |
| Ramachandran statistics |  |
| Favored regions (%) | 98 |
| Allowed regions (%) | 2 |
| Outliers (%) | 0 |

Statistics for the highest-resolution shell are shown in parentheses.

References:

[1] C. Ade, X. Qian, E. Brodszkij, P. De Dios Andres, J. Spanjers, I.N. Westensee, B. Städler, ***Biomacromolecules***, **2022**,

[2] E. Brodszkij, M.J. Hviid, C. Ade, P.S. Schattling, M. Burmeister, S. Szilagyi, N. Gal, C.T. Zhu, X.J. Han, B. Stadler, ***Polymer Chemistry***, **2019**, *10*, 5992.

HAL Amino Acid Sequences:

*Thermoplasma acidophilum* HAL:

ATGATCGAGATTGATGGCCGTAGCCTGCGTGTGGAGGACGTTTACGCGGTGGCGGTTGAATATGATCGTGTTAGCATTAGCGACGATACCCTGAAGGCGGTGGAGGAAAAACACGAAGCGTTTCTGAAGCTGATCAACAGCGGCAAAACCGTGTACGGTGTTAACACCGGTTTCGGCAGCCTGCTGAACGTTCACATCGAGCGTGATCAGGAGATTGAACTGCAAAAGAACCTGATTCGTAGCCACAGCAGCGGTGTGGGCGACTACCTGGAAAACCGTTATGTTCGTGCGATCATGGCGGTGCGTCTGAACAGCCTGGCGGCGGGTTATAGCGCGGTTAGCGCGGATCTGCTGAACATGATGGTGGAGATGCTGAACCGTGACGTTATTCCGGCGGTGCCGAAGTACGGTAGCGTTGGTGCGAGCGGTGATCTGGCGCCGCTGGCGCACATTGGTCTGGCGATGATGGGCGAGGGCAAAGCGTTCTTTGAAGGCCGTCTGATGGACAGCGCGCGTGCGCTGGAAAAGGCGGGTCTGAAACCGTATCAGTTTAAGGAGAAAGAAGGCGTTGCGCTGATCAACGGCACCAGCTTCATGAGCGGCATCCTGAGCATTGCGGTGATGGACGCGCACGATATCCTGGAGAACGCGATTCGTAGCGCGCTGCTGAGCTTCGAAGCGCTGGGTGGCACCAGCAAAGCGTTTACCCCGTGGATTCTGGGCGCGCGTCCGCACCTGGGTCAAGTTGCGATCGGTAACCGTTTTCGTGAGTACCTGACCGGTAGCGACATCGTGAAGCGTGCGGATAGCGTGAAAGTTCAGGACGCGTACACCCTGCGTTGCATCCCGCAAGTTTATGGTAGCGTGGCGGACGTTATTGATTACGTGGAGAACGTTCTGAGCGTGGAAATCAACAGCGCGACCGACAACCCGCTGTTCAACGGCGAGGAAGTGGTTAGCGGTGGCAACTTTCACGGCGAGCCGGTGGCGCTGGCGGCGGATTTCCTGGCGATTGCGCTGACCGACCTGGGCAACATGGTTGAACGTCGTATCGCGCGTCTGGTGGATACCAACCTGAGCGGTCTGCCGCCGTTTCTGACCCCGGACAGCGGTCTGAACAGCGGTTACATGATTCCGCAGTATACCGCGGCGGCGCTGTGCAACCGTAACAAGGTTCTGGCGTATCCGAGCAGCGCGGATACCATCCCGACCAGCGCGAACCAAGAGGACCACGTTAGCATGGGCGCGACCGGTAGCCTGAAGCTGCTGGAAATCATTGATAACGTGCGTTACATCATTGCGATTGAGTATCTGCTGGGTAGCCAGGCGCTGGAGTTCACCGACAAAGGCATGAGCCCGAGCACCCGTAAGATCTACGAAAAAATTCGTGAGAAGGTGGAAAAACTGGACCACGATCGTCCGCCGAGCTTTGATATCGAGACCATTCGTAAAATGATGGACAAGAAGGAGTTCATCAGCGCGCTGCCGTAA

*Alicyclobacillus acidocaldarius* HAL:

ATGACCAAGACCGTGATGATCGACGATATGCCGCTGACCCTGGAGACCTTCTGGGACGTGGTTGAACACCGTATTCCGGTGCAAGTTGGCCCGAACGCGCGTCAGCGTATGCAAGCGTGCAACAGCTACCTGCACGAGACCATCGACAAGGGCGATGTGGTTTATGGTGTGAACACCGGTTTCGGCAAACTGAGCACCGTGGTTCTGAGCCCGACCGACCTGGATGAGCTGCAGCTGAACCTGCTGCGTAGCCACGCGTGCGGTGTTGGCGATCCGTTTCCGGAAACCGTGGTTCGTGCGATGATGCTGCTGCGTCTGGTGAGCCTGAGCCGTGGTTACAGCGGCGTTCGTCCGATCGTGGTTGACACCCTGGCGGCGTGCCTGAACGCGGGTGTGCACCCGGTTATTCCGCAGCAAGGTAGCCTGGGTGCGAGCGGTGATCTGGCGCCGCTGGCGCACCTGGCGCTGGTGCTGATTGGCGAGGGTGAAGCGTGGTGGGAGGGTCAGCGTGTTAGCGGTCGTGAGGCGCTGGAAGCGAGCGGTATCAGCCCGCTGCGTCTGGCGCCGAAAGAAGGCCTGGCGCTGATTAACGGCACCCAAGCGATGACCGCGGTTGGTCTGCTGGCGCACCGTGCGGCGACCTATCTGGCGGAGGCGGCGGATTGCGCGGCGAGCCTGACCATGCAGGCGCTGCGTGGCATTGAAGATGCGTTCGACGATCACCTGCAGCAAGTGCGTGGTTATCCGGAGCAAATCGAAGTTGCGAGCCGTATTCGTGAGTGGACCGAAGGCAGCCGTCTGACCCAGGACAGCCGTACCGCGATCACCTGCGGCAAGGTGCAAGATGCGTACGCGATTCGTTGCGTGCCGCAGGTTCACGGTGCGAGCCTGCGTGTGCTGCGTCAAGTTGAGGACACCCTGAAAATCGAAATGAACGCGGTTACCGATAACCCGCTGCTGTTCCCGGAGGAAGGCCTGATTCTGAGCGGTGGCAACTTTCACGGTCAGCCGGTGGCGCTGGTTATGGACTGCATGAAGATCGCGGTGGCGGAATGGGCGAACATTAGCGAGCGTCGTACCGAACGTCTGCTGAACCCGAGCCTGAACGAGGGTCTGCCGCCGTTTCTGGCGGCGGAACCGGGTCTGCAAAGCGGTCTGATGATCCTGCAGTATACCGCGGCGAGCCTGGTTAGCGAGAACAAAGTGCTGGCGCACCCGGCGAGCGTGGACAGCATTCCGAGCAGCGCGAACCAGGAAGATCACGTTAGCATGGGCACCATTGCGGCGCGTCAGGCGATGCAAATTACCCAGAACACCGCGCGTGTGCTGGCGATCGAGCTGATTTGCGCGGCGACCGCGCTGGAACTGCGTGGTATCGATAACATTAGCCCGGTTGGTAAGAAAGTGGTTGAGTGGATTCGTAACCACGTGGTTAGCGAACTGCGTGACCGTAGCTACAGCCGTGATGTTGAGATTCTGGCGGAAGCGCTGCTGAGCGGTGGCCTGCACAAGACCCTGGCGAACAGCACCAGCTATAGCACCCTGCCGATCCTGCACCTGAACTAA

*Picrophilus torridus* HAL:

ATGATCATTCTGAGCGGCGACGATCTGGACGTGAACAAGCTGTATCGTATCAGCGTTCTGAAGGAGAAAATCGAAATTAGCGAGAGCGCGAAAAACAACGTGCTGCGTAGCCGTAAGAGCCTGGAAAAAATCATTAGCAGCGGCAACGAGATTTACGGTGTGACCACCGGTTTCGGCAGCCTGCTGAACCGTCACGTTGATGAAAAGCTGGAGCTGCAGAAAAACCTGATCCGTAGCCACAGCAGCGGCACCGGCAAGTACCTGAAACCGGAATATGTGCGTGCGGTTATGAGCGTGCGTCTGAACAGCCTGCTGAAGGGTTTCAGCGGCGTTAGCATCGACCTGATTGATCGTCTGATTTTTATGCTGAACAACGACATCATTCCGATCGTTCCGGAGTATGGTAGCGTGGGTGCGAGCGGTGATCTGGCGCCGCTGGCGCACATTGGTCTGGCGATCATGGGTGAAGGCCCGGTTCTGTATAAGAACGACGTGCGTGATAGCAACGAGGTTTTCCGTATCTTCAACATCGAAAAGTACGAGTTTAAGGAGAAAGAAGGCGTGGCGCTGATCAACGGCACCAGCATGATCACCGGCATTCTGGGTATCGAACTGTATCGTAGCATGAACCTGATTAAGCACGCGCTGATCAGCAGCATGATGGCGTTCGAAGCGCTGAGCGGCACCGACAAAGCGTACCGTGAATGGGCGATTGCGAGCCGTCCGCACAACGGTCAAATCACCATTGCGGACAACCTGCGTTTTATTCTGAAGGATAGCGAAATCGTGAAAAACGCGAACAAGATCAAAATTCAGGACGCGTACACCCTGCGTTGCATCCCGCAAGTTTATGGCGCGGTGCTGGATGCGATTAAATACAGCATCAACGTGGCGAACACCGAGATGAACAGCGCGACCGACAACCCGCTGGTTTACAACGACGATTATATTAGCACCGGCAACTTCCACGGTGAACCGGTTGCGCTGGCGGCGGACTTTAGCGCGATTGCGCTGACCGATCTGGGCAACATGATTGAGCGTCGTATCGCGCGTCTGGTGGATACCAACCTGAGCGGTCTGCCGCCGTTCCTGGTTAACAACTACGGCATTAACAGCGGTTACATGATCCCGCAGTATACCGCGGCGGCGCTGTGCAACATGAACAAGATTCTGAGCCACCCGGCGAGCGCGGACAGCATCCCGACCAGCGCGAACCAAGAGGATCACGTGAGCATGGGTGCGAACAGCGCGCTGAAGCTGATCAAAATTCGTGAAAACATTGAGCGTATCATTGCGATTGAATATCTGATCGCGGCGCAGGCGCTGGAGTTCAAGAAATATAACACCAGCAACTTCATCGACATGGTTTACAAGAAAATTCGTAGCCGTATCGACCAACTGGAAAACGATCGTCCGCCGTACATCGACATTGAAAAAATTCTGGAGATGATGAACAACGATCTGATGGAGTTTATCAACAGCATCCGTATTTAA

*Kosmotoga olearia* HAL:

ATGGTTATCATTAACGGCAACGATCTGACCGTGGAACAAGTTTACAGCGTGGCGTTTCACGGTGAGGAAGTGCGTATCGCGGAAAGCACCCTGGACGAGCTGAAGAAAAAGCGTCTGTTTCTGGAGGAAATGCACAACAAGGATGTTATCTATGGTATTAACACCGGTTTCGGCATCCTGGCGGACAAGCGTATTAGCGACGATGACCTGGAGAAACTGCAGGTTAACATTGTGCGTAGCCATGCGGCGGGTGTGGGTGAACCGCTGAAGACCGAGCTGGTTCGTGCGATTATGCTGGTGCGTGCGAACAGCCTGTGCAAAGGCTACAGCGCGGTTCGTCCGGAAGTGGTTCAGCAACTGGTGAACTTTCTGAACAAGGGTATCGTTCCGATTGTTCCGGAGCAAGGTAGCGTTGGTGCGAGCGGTGATCTGGCGCCGCTGGCGCACATTGCGCTGGCGCTGATTGGTGACGGCGAAGTGTTCCACAACGGCCGTAAAGAAAAGGCGCTGGAGGCGATCCAGGCGGAAGGTCTGAAACCGCTGACCCTGAAAACCAAAGAGGGTCTGAGCCTGCTGAACGGCACCGCGTTTATGGCGGGTATCGGCGCGTGCAGCGTTCACATTGCGGAAAAGATGTTCGACAAAGCGATCCTGGTTGCGGCGATGAGCGTGGATGCGCTGATGGGCAGCACCAGCCCGTTTGACCCGCGTATCCAGGAAGCGCGTCCGCATCCGGGTCAAAAGTACGTTGCGAAAAAGCTGCGTGAGTATCTGGAAGGTAGCGAGATCCGTCGTAGCCACCTGCACTGCGATAAAGTGCAGGACGCGTACACCCTGCGTACCATTCCGCAAGTTTACGGTGCGGTGTATGATACCCTGCAGTATGTTAAGAGCGTGATCACCCGTGAGATTAACAGCGCGACCGACAACCCGCTGATCTTCGATAACGGCGACGTTATTAGCGGTGGCAACTTTCACGGTGAACCGATCGCGCTGGTGCTGGATTTCCTGAGCATTGCGCTGACCGACATGGCGAACATGATGGAGCGTCGTGTTGATCGTCTGGTGAACCCGAAGCTGAACAACTTCCCGGCGTTTCTGACCCGTGGCAAAGAAGGTCTGAACAGCGGCTACATGATCTGGCAGTATACCGCGGCGGCGCTGGCGAGCGAGAACAAGACCCTGGCGCATCCGGCGAGCGCGGATACCATCCCGACCAGCGGTTTTCAGGAAGACCATGTTAGCATGGGCGCGTGGGGTGCGCGTAAACTGTGGAAGATTCTGAAAAACTGGAGCAACATCCTGGCGATTGAGACCGTGCTGGCGTACCGTGCGCTGAGCTTCCGTAAGCCGAAAAAGAGCGGCAAAGCGATCGAAGGTTTCTTTAAGGAGCTGAGCAACATTCTGGAGGAACACGTGGAAGACCGTTATTTCGGTAAAGAGTTTGCGGATGCGCGTGACTTCCTGCTGAAGAGCCAAGGCCTGAAAGGTTTTTAA

*Acidilobus saccharovorans* HAL:

ATGAAGGTGTGCGCGGGTCAGCGTCTGACCGTTGAGGACATCTGCAACGTGGCGGTTAACAGCCCGGTTAGCATGTGCCAGGAGAGCCTGCAACTGCTGAGCCGTAGCCGTAAGGCGTACGAAGCGGGTGCGAGCCTGGGCAAAGTGTACGGTTATAGCACCGGTCTGGGCGCGCTGGCGAGCGCGGAGGAAGGCACCTGGCGTGGTCGTGAGGCGCAGGTTCTGCGTGAACATGACCTGGGTATTGGTCCGGAGGCGCCGCGTGAAGTGGTTCGTGCGGCGATGCTGGTGCGTGCGAGCCAGCTGGCGCAAGGTTTTGCGCCGGTGCGTCCGGAAGTGGCGCAACGTCTGGTTGACAGCCTGAACTACGATATTGTGCCGGCGGTTAGCCTGGAAGGTAGCGTGGGTGCGAGCGGTGATCTGGCGCCGCTGGCGCGTATCAGCCGTTGCATTTATAGCGGTGAAGGTCAAGCGACCGTTCGTGGCCGTCGTCTGAGCTGCGGCGAGGCGCTGGAAGCGGCGGGTCTGAAACCGCTGGAGCTGGGTCCGGGCGAAGCGCTGGCGATCATTAACAGCAACGCGTTCAGCGTTGCGATGGCGAGCCTGGGCGTTTGCGCGAGCCTGCGTCTGCTGGGCGAGAGCCTGAACGTGATGAGCGAGACCCTGGAAGTTACCGGCTGCAACCCGCAACACTTTAGCACCGCGGTGGCGAACAGCAAGCGTCTGGAAGGTGTTAAAGACGTGGTTGCGAGCCTGAAGGTGGATTGCCAGCGTAGCCCGCGTCTGCAAGACCCGTACTGCATCCGTTGCGTGCCGCAGGTTTATGGCAGCGCGCTGGAAGTGCTGCGTTTCGCGGCGAAACTGCTGGAGGCGGAGGCGTACAGCAGCAGCGACAACCCGTTCGTGTACAACGATAGCGTTTATCATGCGTGCAACTTTCATGCGAGCGCGGCGGGTATGGCGAGCGACATGGCGAAGGTGGCGCTGGCGCACGTTGGTAACATGATTGAACGTCGTACCGCGCACCTGCTGAGCAGCGCGACCACCGGTCTGCCGGATTTTCTGGCGGTTAAGGGCACCGTGGTTGGTGCGATGATCTACCAGTATACCGCGGCGAGCCTGGCGGCGAAACTGCGTCAACTGGCGTCTCCGGGTAGCGTGCACAGCATTCCGACCAGCGGTCTGCAGGAAGACGTGGTTAGCATGGCGCCGAACAGCAGCTATGAACTGCTGCGTAGCACCGCGGTGCTGGCGCGTCTGATTGCGGTTGAGGATGCGCTGGCGAGCCAAGCGAGCAAAGTTGCGCGTGGTCTGCCGCTGGACGATCCGAAGGATGCGCTGGAGCGTAGCCTGGCGAAAGTGGTTGGCGAAGCGGGTCTGAGCGGCCTGAGCTTTATTGTGAACCTGGCGTAA

*Caldisphaera lagunensis* HAL:

ATGCTGTGCAAGAACGAGATCAGCCTGGACGATTTCTTTAAGATTCTGTTCAGCAACGAAAAAATCAGCATTTGCGAGGAACGTCTGAACAAGATCAAACGTAGCCGTGAGGTGTACGAGACCGAAGGTAAGAAAGGCAAGGTTTACGGCTATAGCACCAGCCTGGGTGCGCTGCTGAACAAAGAAAACGCGTACACCGAGGAACGTGAGAAGATCATTCTGAAAGAACACGACACCGGTATCGGCAAGAACGTGCCGTATCTGCTGGTTAAAGCGGCGATGCTGGTGCGTCTGATCCAGCTGAGCAACGGCAACAGCCCGATTCGTCAAGAGGTGGTTAACCGTCTGATCGACGCGATTAACTACGATATCATTCCGACCGTGAGCATCCTGGGTAGCGTTGGTGCGAGCGGTGACCTGGCGCCGCTGGCGCGTATTTTCCGTTGCATCATTTATGGTGAAGGCAGCGCGTACTATAAGGGTAAAGTGGTTAACTGCGTGGATGCGCTGAAGGAGAAAGGCCTGAACGTTATGAACCTGATTCCGGGTGAAGCGCTGGCGCTGATTAACAGCAACGCGTTCAGCACCGCGTTTAGCATCATTAACGCGTACATCACCGAGCGTCTGGCGTATGCGAGCCTGGACATCGCGAAGAAAAGCCTGAAAATTACCGGCTGCAACAACGAGCACTTTAGCGATGCGGTGTACGAAACCAAGAAACAGATGGGTGTGAAGAAAGTTATCGACATGCTGAACAACAGCTGCGAGGAAACCCCGCGTCTGCAGGACCCGTACAGCATCCGTTGCCTGCCGCAAATTTATGGTGCGCTGTTCGACACCCTGGAGTTTAGCAAGAGCATTCTGAAAAACGAAATGAACAGCAGCAGCGATAACCCGATCATTTACGAAAACAAGGTTTATCACGCGTGCCAATTCCACGCGATCTATAGCGCGCTGATTAGCGACTTTCTGAAAATCGCGATCATTCCGGTGATGAACAGCATTGAGCGTCGTATCAGCCAGATTCTGGACGGCAAGATCAACCGTGTTAACGATTTTCTGCTGAAAGAAAACAGCGTGGTTGGTGCGATGATCATGCAATACACCAGCGCGGCGCTGAACGCGTATAGCCGTGGCAGCAGCATTAGCATGAGCAGCCACAGCGTGCCGACCAGCGGCACCCAGGAAGACATCGTTAGCATGGCGCCGAACAGCAGCATTGAACTGCTGAAGATGAACAGCCTGTTCCTGAAAATCATTGCGGTTGAGCTGGCGCTGTACAAGTACTATAACAGCAAAGACCTGCCGGACCCGCGTATCCTGCTGGAGGAGAGCTTCAGCGAGATCATTAACAACTACAACCTGAAGAACTTCAGCGAATTTCTGAACTATTTCTTTTAA
